# Thymic Program Directing the Functional Development of γδT17 Cells

**DOI:** 10.3389/fimmu.2018.00981

**Published:** 2018-05-08

**Authors:** Youenn Jouan, Emmanuel C. Patin, Maya Hassane, Mustapha Si-Tahar, Thomas Baranek, Christophe Paget

**Affiliations:** ^1^INSERM, Centre d’Etude des Pathologies Respiratoires (CEPR), UMR 1100, Tours, France; ^2^Université de Tours, Tours, France; ^3^Service de Médecine Intensive Réanimation, Centre Hospitalier Régional Universitaire de Tours, Tours, France; ^4^Division of Radiotherapy and Imaging, Targeted Therapy Team, The Institute of Cancer Research, London, United Kingdom; ^5^Department of Biochemistry and Molecular Genetics, Faculty of Medicine, American University of Beirut, Beirut, Lebanon

**Keywords:** γδT cells, innate immunity, interleukin-17A, thymus, transcription factor, development

## Abstract

γδT cells comprise a unique T cell sublineage endowed with a wide functional repertoire, which allow them to play important—sometimes opposite—roles in many immune responses associated with infection, cancer, and inflammatory processes. This is largely dependent on the existence of pre-programmed discrete functional subsets that differentiate within the thymus at specific temporal windows of life. Since they represent a major early source of interleukin-17A in many models of immune responses, the γδT17 cell population has recently gained considerable interest. Thus, a better dissection of the developmental program of this effector γδT subset appears critical in understanding their associated immune functions. Several recent reports have provided new exciting insights into the developmental mechanisms that control γδT cell lineage commitment and differentiation. Here, we review the importance of thymic cues and intrinsic factors that shape the developmental program of γδT17 cells. We also discuss the potential future areas of research in γδT17 cell development especially in regards to the recently provided data from deep RNA sequencing technology. Pursuing our understanding into this complex mechanism will undoubtedly provide important clues into the biology of this particular T cell sublineage.

Interleukin-17 (IL-17) is a highly conserved cytokine in vertebrates that plays a critical role in host homeostasis and immune response to pathogens especially at barrier sites (1, 2). Recent evidence indicates that IL-17 also emerges as a key contributor in immunity beyond the scope of infection, such as inflammation and cancer (3, 4). Given the pivotal role of lymphoid cell-derived IL-17 in orchestrating immune responses, its cellular sources have been extensively searched over the last decade. Initially believed to be mainly produced by conventional CD4^+^ T (Th17) cells (5, 6), the discovery of innate and innate-like lymphocytes endowed with potent capacities to produce IL-17 (7) suggests that this cytokine is well poised at the border between innate and adaptive immunity. These populations include γδT cells (8), natural killer T (NKT) cells (9), mucosal-associated invariant T (MAIT) cells (10), and group 3 innate lymphoid cells (ILC3) (11). Among those, γδT cells have been demonstrated to be the main contributors in IL-17 production in many settings, such as infection, autoimmunity, and cancer. Here, we discuss the recent advances on our understanding of IL-17-producing γδT (γδT17) cell biology with a particular emphasis on the transcriptional road maps that drives their innately “pre-programmed” effector fate.

## Characteristics of γδT17 Cells

Mouse γδT cells consist of a heterogeneous population of thymus-derived T lymphocytes characterized by distinct functional properties (e.g., cytokine profile and/or cytotoxic properties) and tissue distribution (12). Within this subset diversity, γδT17 cells can be defined based on their T cell receptor (TCR) repertoire usage and surface markers. Thus, γδT17 cells are almost exclusively restricted to γδT cells expressing either a Vγ6 or a Vγ4 TCR (N.B.: The Heilig and Tonegawa’s nomenclature (13) has been used in this review) (Table 1). In addition, many cell surface antigens (Ags) have been shown to distinguish γδT17 cells and can be defined as CD27^−^, NK1.1^−^, IL-7Rα^high^, IL-18R^high^, CD122^−^, and CCR6^+^ cells (14–16). γδT17 cells mainly establish residency at barrier sites including lung, skin, vagina, and oral cavity. However, they can recirculate in particular pathological situations including infections and cancer (17). The migratory capacity of γδT17 cells is regulated by the chemokine receptors CCR2 (during inflammation) and CCR6 (at homeostasis) (18). This preferential location at barrier sites might indicate a preferential interplay between γδT17 cells and the endogenous flora (19) as exemplified by the strong reduction in frequency of lung resident γδT17 cells in germ-free mice (20). Interestingly, while Vγ4^+^ γδT17 cells can be detected in the gastrointestinal tract (21), we and others failed to detect Vγ6^+^ γδT17 cells in this tissue in adult mice under steady-state condition (20, 22). This might suggest that the nature and/or diversity of commensals in the various mucosa could differentially influence the maintenance of γδT17 cell subsets.

**Table 1:** Origin, tissue distribution, and TCR repertoire of γδT17 cells.

Subset	Windows of development	Steady-state tissue distribution	Origin	V(D)J diversity
Vγ1^+^	Mainly perinatal (day 3–8)	Barrier sites and lymphoid tissues	Natural	Intermediate to high
Vγ2/3^+^	Late embryonic and perinatal (from E17 to day8)	Barrier sites and lymphoid tissues	Natural	–
Vγ4^+^	Late embryonic and postnatal (E18 onward)	Barrier sites and lymphoid tissues	Natural: for Vγ4^+^ T cells of fetal origin including at least the Vγ4Vδ5 subset	Invariant for natural γδT17: Vγ4Jγ1/Vδ5Dδ2Jδ1
			Inducible: after Ag encounter in the periphery without extensive clonal expansion	Intermediate to high for inducible γδT17
Vγ5^+^	Embryonic only (from E13 to E16)	Epidermis	Reprogrammed: unknown mechanism (TCR?)	Invariant: Vγ5Jγ1Cγ1/Vδ1Dδ2Jδ2
Vγ6^+^	Embryonic only (from E14 to birth)	Barrier sites at the exception of the gut	Natural	Invariant: Vγ6Jγ1Cγ1/Vδ1Dδ2Jδ2

*E, embryonic day; V, variable gene segment; D, diversity gene segment; J, junction gene segment; Ag, antigen; TCR, T cell receptor*.

γδT17 cells are characterized by their ability to promptly produce copious amounts of IL-17A/F, IL-22, IL-21, and GM-CSF (8, 23–25). This rapid capacity to produce these cytokines can be mainly attributed to their innate-like feature. Despite expressing a fully functional rearranged TCR, γδT17 cells can respond to activating cytokines (IL-1β, IL-23, and IL-18) even in absence of concomitant TCR engagement (8, 16). However, TCR ligation on naive γδT17 cells has been shown to license them by increasing activating cytokine receptor expression (e.g., IL-1R1 and IL-23R) and thus rendering them permissive to “innate” stimulation (26). The nature of these Ags is yet to be determined. Notably, the unprocessed form of the red algae protein phycoerythrin has been shown to interact with a small proportion of naive γδT cell TCRs irrespectively of their TCR repertoire (26). Even if the physiological relevance of phycoerythrin in the biology of mammalian cells is difficult to conceive, it is tempting to speculate that structurally related Ags could be relevant in the general selection and licensing of γδT cells. In addition, the fact that a single Ag can be recognized by various γδTCRs harboring distinct CDR3 regions is reminiscent with the NKT cell biology (27) and can suggest the existence of a restricted conformational “hot-spot” comprising few amino acid residues in CDR3 regions responsible for γδT cell antigenicity in mice. This structural basis for Ag recognition by innate-like T cells might have been conserved all through the evolution from jawless vertebrates (28).

Despite leaving the thymus with a pre-programmed effector fate, γδT17 cells have been shown to conserve a certain degree of plasticity in the periphery. This characteristic originates from an epigenetic regulation program for specific genes in γδT17 cells such as Dickkopf-related protein 3 (29). Thus, along with IL-17, γδT17 cells can also produce interferon (IFN)-γ under inflammatory conditions. The biological relevance of this plasticity has been revealed in various settings including *Listeria monocytogenes* infection (22). In this later model, long-lasting accumulation of Vγ6^+^ IFN-γ/IL-17 double producers was observed within intestinal lamina propria (22, 30). Since Vγ6^+^ γδT17 cells were reported to be absent from the gastrointestinal tract at steady-state (20), it is possible that the combination of the γδT17 cell epigenome and local environment modifications under this inflammatory condition favors their homing and survival in the gut tissue. On the other hand, it is interesting to mention that IFN-γ-producing pre-programmed γδT cells (γδT1) do not possess the capacity to produce IL-17 (31). However, a small proportion of epidermal γδT1 (e.g., Vγ5^+^) cells has been demonstrated to produce IL-17 *in vivo* upon skin wounding (32). The molecular determinants involved in giving rise to this cytokine production capacity are currently unknown but seem to rely on TCR signaling (33).

The existence of γδT17 cells in humans is still a matter of debate (34). Actually, the thymic program of γδT cells in humans seems to differ from the one described in mice (35). Most of the data available suggest that human γδT cells might not be “innately” programmed to produce IL-17 during their thymic development but rather acquire this capacity under inflammatory conditions once in the periphery akin to CD4^+^ Th17 cells. Thus, circulating Vγ9Vδ2^+^ [using Lefranc’s nomenclature (36)] T cells from adult healthy donors produce no or little IL-17 (37) except under complex stimulatory protocol including both activating cytokines and TCR engagement (38). However, it is important to mention that purified Vγ9^+^ γδT cells from cord blood seem more prone to produce IL-17 (37, 39, 40) that could suggest an embryonic origin for human γδT17 cells similar to the murine situation. It is, therefore, possible that these putative γδT17 cells occupy particular niches of the body that render them difficult to assess under homeostatic conditions. Murine pre-committed γδT17 cells are often characterized by the expression of an almost clonal TCR (41). Thanks to next-generation sequencing, the existence of clonal TCR-expressing γδT cell subsets in humans has recently emerged. Upon cytomegalovirus reactivation, a recent study demonstrated the massive proliferation of diverse γδT cell clones in patients after allogeneic-hematopoietic-stem-cell transplantation (42). However, the *TRG* and *TRD* sequences of these clones were not shared among individuals at the nucleotide level (42). In addition, analysis of the human Vδ1^+^ T cells in healthy adults indicates that this repertoire is dominated by few private clonotypes (43). Determining the cytokine profile of these clones will be helpful to better appreciate the existence and origins of γδT17 cells in humans. Whatever the mechanisms that drive their emergence in humans, the capacity of human γδT cells to produce IL-17 has been demonstrated in various immune responses including infection, cancer, and autoimmunity (38, 44–46).

## Development of γδT17 Cells

### Dealing With the Concept of “Innate/Natural” vs “Adaptive/Inducible” Origins of γδT17 Cells

Mouse γδT cells develop in a standardized manner by sequential waves that can be conveniently followed based on their Vγ chain usage (47). This process starts during embryonic life from day 13 (E13) onward. The first wave is exclusively constituted of the IFN-γ-producing Vγ5^+^ cells and lasts for about 4 days. This is shortly followed by a developmental wave of “natural” IL-17-producers comprising both canonical Vγ6^+^ (from E14 to birth) and restricted subsets of Vγ4^+^ (E18 onward) (48, 49). Around birth, IFN-γ-producing Vγ1^+^ and Vγ4^+^ subsets start to develop along with the IL-4/IFN-γ-double producers Vγ1^+^Vδ6^+^ subset. After birth, developing γδT cells mainly exhibit a naive uncommitted profile (47).

According to this scheme, natural γδT17 cell development is believed to be restricted to the gestational period. To support this notion, Haas and colleagues demonstrated that transplantation of bone marrow from IL-17-competent mice into lethally irradiated *Il17af*-deficient adult recipients failed to induce γδT17 cell development (49). Partial γδT17 cell development in the thymus could be achieved in reconstituted *Il17af*-deficient neonate recipients but failed to give rise to γδT17 cells in the periphery. In addition, inducible expression of *Rag1* in T cell precursors in adult mice did not restore *de novo* generation of γδT17 cells (49). Likewise, CCR6^+^ IL-17-producing dermal γδT cells failed to reconstitute 8 weeks after bone marrow transplantation unless the mice received an additional transfer of neonatal thymocytes (33). Surprisingly, the same lab reported the presence of Vγ4^+^ CCR6^+^ (but not Vγ4^−^ CCR6^+^) γδT17 cells in lymph nodes of recipient TCRδ^−/−^ mice following bone marrow transplantation in absence of neonatal thymocytes at 12 weeks post-grafting (50). The basis for this difference remains unclear but one can argue that, in the 12 weeks model, authors have reconstituted “inducible” γδT17 cells only. Several studies have also reported that the peripheral pool of γδT17 cells decreased with age (51, 52), which further support the embryonic origin of natural γδT17 cells.

Despite suggesting a strict favorable temporal window for natural γδT17 cell development during fetal life, these studies also raised some additional interrogations. Is this developmental model imposed by intrinsic (nature of γδT precursors) or extrinsic (embryonic vs adult thymic environment) factors? Zúñiga-Pflücker’s team recently started to provide an answer to this question. Culture of γδTCR-transduced fetal or adult hematopoietic precursors with OP9-Delta-like protein 4 (Dll4) cells led to the development of γδT cells with IL-17 production capacity in a similar manner (53). Thus, these data suggest that adult progenitors conserve their intrinsic capacity to develop as γδT17 cells once in an appropriate environment. However, since the authors used a clonal TCR (Vγ4Vδ5) (54) for transfection, it cannot be excluded that in addition to the favorable environment, the nature of the TCR expressed plays a role in the IL-17 effector fate, in particular regarding the recent discovery of clonal Vγ4Jγ1/Vδ5Dδ2Jδ1 T cells with a strongly biased IL-17-producing profile (55). In addition, recent studies have shown that adult peripheral γδT cells (from adult bone marrow-derived precursors) can convert into “induced” γδT17 cells upon inflammatory conditions (56, 57). Both studies highlighted a critical role for the cytokines IL-23 and IL-1β, and to a lesser extent TCR signaling, in this process (56, 57). Importantly, the potential to give rise to inducible γδT17 cells seems to be restricted to the IL-2Rβ^−^ γδT cell subset (57). To further illustrate this peripheral polarization capacity, Buus and colleagues recently provided a RNA-Seq analysis of adult γδ thymocytes indicating IL-17 potential in certain subsets including notably IL-2Rα^+^ Clec12A^+^ Vγ1^+^ and Vγ4^+^ cells (58).

This situation illustrates the recent concept of “natural” vs “inducible” γδT17 cells (48). While “natural” (Vγ6^+^ and Vγ4^+^ subsets) γδT17 cells are committed to this effector fate during their embryonic/perinatal thymic developmental program, “inducible” γδT17 cells stem from naive (Vγ1^+^ and Vγ4^+^ subsets) γδT cells within the periphery upon inflammatory conditions through cytokine, and/or Ag recognition akin to conventional CD4^+^ Th17 cells (26). Thus, it is tempting to view “natural” γδT17 cells as innate-like T cells whereas “inducible” γδT17 cells can rather be considered adaptive. This is reminiscent with the situation in the αβ lineage comprising innate-like T17 (NKT17 and MAIT17) cells and adaptive conventional Th17 cells.

### Thymic Molecular Determinants of γδT17 Cell Effector Fate

In this section, we will review the thymic determinants that drive γδT cell differentiation into an IL-17 effector fate by deciphering how “natural” IL-17-committed γδT cells emerge from the rest of the γδT cell compartment. Before commitment into γδT17 cell sublineage, thymic precursors have to first undergo a bifurcation into αβ or γδ lineages. Mechanisms driving this initial dichotomy are beyond the scope of this review, but it is worth mentioning that the strength of TCR engagement in thymocytes emerges as a driving force in this process [see Ref. (53, 59) for reviews]. However, requirement for TCR ligation in IL-17-commited γδT cell differentiation is still an intense matter of debates that will be discussed later. Commitment toward the γδT lineage happens at double negative (DN)2 and DN3 stages (60, 61). Interestingly, the effector fate of “natural” γδT cell subsets (IFN-γ- vs IL-17A-producing) appears to be already predetermined at this stage. Thus, commitment to “natural” γδT17 cells exclusively arises from the late DN2 stage in a B cell leukemia/lymphoma 11b-dependent manner (46).

Further differentiation into the IL-17 effector fate is a complex and highly dynamic process involving multiple molecular and cellular interactions. For the salve of clarity, we distinguish here the (1) extrinsic factors (e.g., thymic environmental cues) and (2) intrinsic factors [e.g., intracellular signaling pathways and transcription factors (TFs)] that tune γδT17 precursors into mature γδT17 cells.

#### Extrinsic Factors

**Figure 1:**
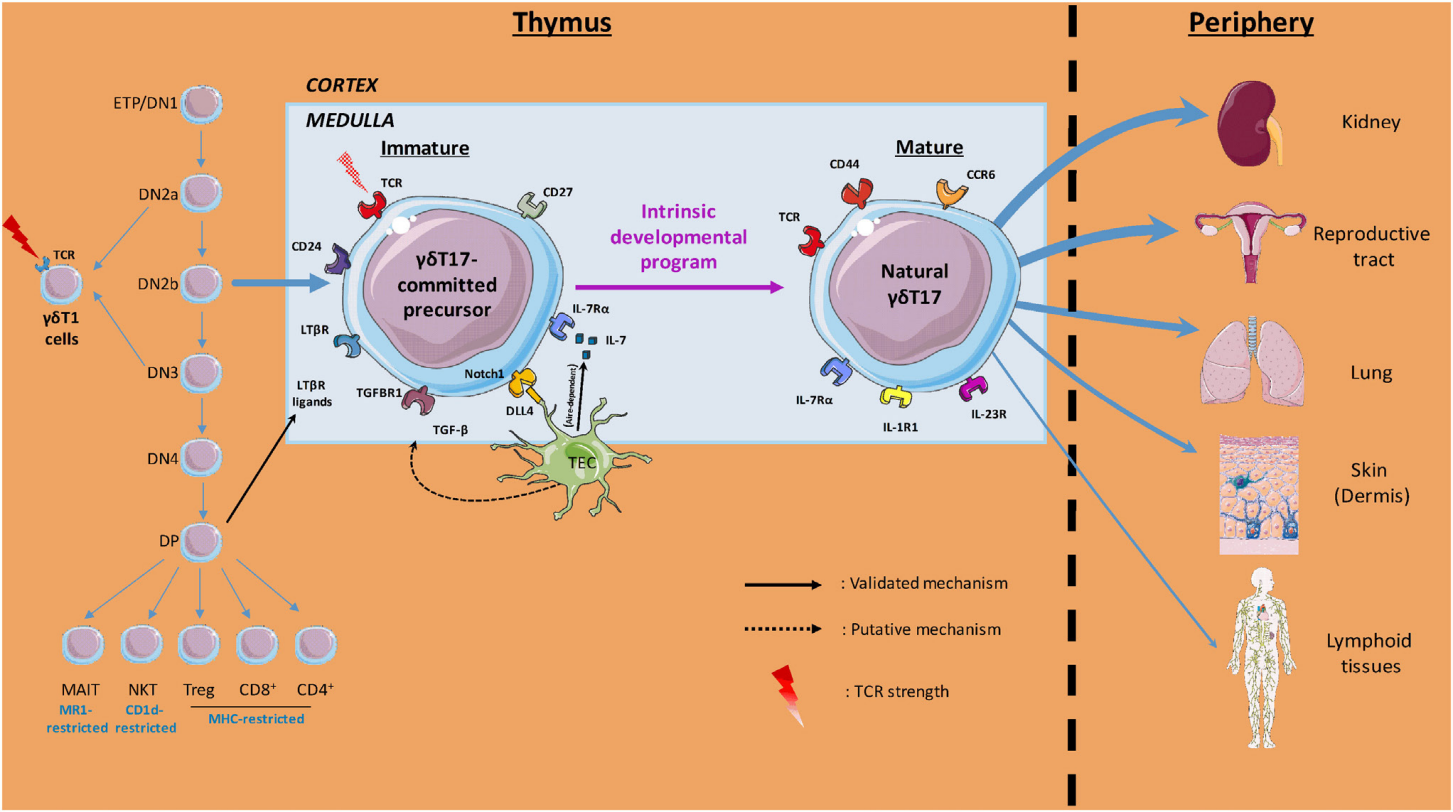
Overview of thymic γδT17 cell ontogeny. Initial intrathymic pathways leading to pre-committed γδT17 cells divergence from other T cell lineages are illustrated. The thymic (cortical and medullary) environmental cues involved in γδT17 cell effector fate and the preferential peripheral niches of mature γδ17 cells are also depicted. Labels indicate the cells, soluble factors, proteins, surface markers, and T cell receptor (TCR) signal strength involved in the γδT17 cell program of differentiation.

Molecular and cellular players within the thymic microenvironment are crucial for acquisition of γδT cell effector fate (Figure 1). Anatomically, precursors migrate from the cortex to the medulla during this process, where they receive multiple signals that dictate their differentiation. In this three-dimensional environment, it is important to keep in mind that time should be considered as a fourth dimension when deciphering the “natural” γδT17 cells ontogeny.

#### *Cytokines* 

Many cytokines have been reported to directly or indirectly regulate thymic γδT17 cell differentiation/development.

IL-7 is a critical and non-redundant cytokine in lymphopoiesis (62). IL-7Rα-deficient mice completely lack γδT cells (63, 64) partly due to the role of IL-7 in V-J recombination of TCRγ genes (63, 65). Its specific role in survival and proliferation of γδT17 cells in peripheral tissues under homeostatic and pathological situations has been demonstrated (37, 66, 67). Even if this question has never been directly addressed, many studies imply a requirement for IL-7 in the proper natural γδT17 cell development. First, in a model of conditional abrogation (RBP-Jκ) that precludes IL-7Rα (CD127) expression, but in which the initial generation of γδ precursors (DN2 and DN3 stages) is maintained, the pool of both thymic and peripheral γδT17 cells is markedly reduced (68). Second, addition of recombinant IL-7 in fetal thymic organ culture from E16 thymus promotes γδT17 cell expansion over IFN-γ-producing γδT cells (37). Understanding whether IL-7 directly participates in the differentiation program or solely in the post-differentiation expansion of γδT17 cells will require further investigations. In line with the favorable temporal window for γδT17 cell development around birth, it is important to mention that thymus is particularly enriched for IL-7 [especially in thymic epithelial cells (TECs)] in neonates and its presence declines with age (69, 70).

Akin to conventional αβ Th17 cells, TGF-β1 has also been proposed to participate in γδT17 cell development (71). In addition, this report indicates that some other Th17-driving cytokines, such as IL-23 and IL-21, appear to be dispensable. However, since the authors have performed their investigation in 11-day-old mice, a time at which natural γδT17 cells already egressed the thymus, it is difficult to precisely evaluate the contribution of TGF-β1 in natural γδT17 cell differentiation.

The role of IL-6 in γδT17 cell development is somehow controversial. While several studies indicated that IL-6 deficiency did not influence γδT17 cell homeostasis and cytokine production capacity (71, 72), others reported a defect in γδT17 cells in both thymus (73) and peripheral organs (74). Thus, the contribution of IL-6 in natural γδT17 cell differentiation remains unclear. In addition, mRNAs for IL-6Rα (CD126) are barely expressed on both immature and mature thymic natural γδT17 cells (from the ImmGen database). However, it cannot be firmly excluded that IL-6 influences the thymic microenvironment to support γδT17 cell differentiation as previously hypothesized (73).

Thus, this indicates that the cytokine network within the thymic microenvironment has to be tightly regulated in a time-dependent manner to allow natural γδT17 cell differentiation.

#### *Thymic* *Epithelial Cells*

Akin to other thymocytes, interaction with TEC is likely critical in γδT17 cell differentiation, even though few experimental data are currently available. Thus, medullary (m)TEC has been implicated in regulating Vγ6^+^ γδT17 cell development through the TF autoimmune regulator (Aire) (69). In *Aire^−/−^* mice, IL-7 production is up-regulated in mTEC, and this is accompanied by an overproduction of Vγ6^+^ γδT17 thymocytes. Interestingly, other subsets of natural γδT17 cells, especially Vγ4^+^ subsets were not affected by specific Aire deletion in mTEC (69). This feature also indicates that the various natural γδT17 cell subsets probably require different signals to develop.

Cortical (c)TEC has also been recently shown to control γδT17 cell development. Using a mouse model with specific cTEC ablation, Nitta and colleagues observed a strong dysregulation in the proportion of natural γδT17 cell subsets (75). Specifically, absence of cTEC skewed the γδT17 TCR repertoire toward Vγ6 expression at the expense of the Vγ4^+^ T cell subset during the postnatal period while the proportions of Vγ6^+^ and Vγ4^+^ γδT17 cells remained normal during embryonic life. Authors hypothesize that in their mouse model, the postnatal thymic microenvironment resembles to the fetal microenvironment, which in turn favors the Vγ6^+^ subset. In addition, since they have been proposed to be a prime source of TGFβ (76), cTEC could also participate in thymic development of γδT17 cells through this mechanism (71).

The importance of Dll4, a Notch ligand expressed by TEC (77) has also been proposed in γδT17 cell differentiation (78). In a co-culture model of E15 thymocytes with stromal cells, absence of Dll4 expression by stromal cells led to an abrogation in γδT17 cell development (78). This phenotype indicates that Dll4 is likely to be a common factor for the differentiation of all natural γδT17 cell subsets.

Finally, a recent study proposed that signaling through NF-κB-inducing kinase (NIK) in TEC is essential for the generation of a fully functional pool of γδT17 cells (79). However, the molecular factors regulated by NIK in TEC are yet to be determined. Understanding the NIK-dependent pathways in TEC will certainly provide important clues in the TEC-γδT17 precursor interaction mechanisms that drive γδT17 cell effector fate.

Altogether, the fetal and perinatal thymic environment offers a temporal window of opportunity for γδT17 cell differentiation. However, the available literature indicates the requirement for differential factors according to the subset of naturally occurring γδT17 cells. This might somewhat rely on the intrinsic nature of the γδT17 precursors. It can also be hypothesized that these precursors (Vγ6^+^ and Vγ4^+^) require timely expressed TCR ligands in the thymic environment. However, no host-derived Ags have been proposed to date to participate in γδT17 cell differentiation.

#### Intrinsic Factors

##### A Requirement for TCR Ligation: Still an Open Question?

Beyond its importance into γδ lineage commitment, TCR signal strength is also involved in the functional maturation of γδ-committed thymocytes. Specifically, TCR signal strength drives the IL-17- vs IFN-γ-producing γδT cell dichotomy. However, it appears difficult to clearly attribute a specific strength to a specific effector fate. While the literature tends to demonstrate consensually that a strong TCR signaling in γδ thymocytes drives their commitment toward a Th1-like effector fate (31, 80–82), the situation in γδT17 cell differentiation remains highly debated.

Chronologically, a first set of data suggested that γδT17 cell differentiation occurred in the absence of TCR cognate ligands (80). However, (1) this study used adult thymocytes and focused on peripheral organs that are weakly if not populated with natural γδT17 cells and (2) it cannot be excluded that ligand-independent TCR signaling plays a part in this model. Therefore, these results are likely to provide specific information about the requirement of TCR signals for inducible γδT17 cells. In this sense, these data perfectly fit with the concept that inducible γδT17 cells egress the thymus with a naive uncommitted profile and need further encounter with peripheral Ags to gain their capacity to produce IL-17. Few years later, the lab of Adrian Hayday highlighted the butyrophilin-like molecule Skint-1 as a molecular determinant in Th1-like effector fate of Vγ5^+^ T cells (31). Interestingly, in absence of Skint-1, the differentiation of Vγ5^+^ T cells resulted in the generation of cells displaying a phenotype of natural γδT17 cells (31). Indeed, Skint-1 engagement in Vγ5^+^ thymocytes induces the upregulation of TCR-dependent genes that subsequently repress the transcriptional differentiation program of natural γδT17 cells. In line, Pennington and colleagues recently demonstrated that differentiation of E15 γδ thymocytes in presence of an anti-TCRδ mAb (GL3) blunted their commitment toward a γδT17 cell profile (82). Altogether, weak or no TCR signals seem required to allow proper γδT17 cell development. Thus, the developmental program of natural γδT17 cells appears to be a TCR Ag-free process acquired by “neglect.”

On the other hand, mice presenting a reduced function in the TCR proximal signaling kinase ZAP-70 displayed a reduced pool of both IL-17A-producing Vγ6^+^ and to a lesser extent Vγ4^+^ T cells in neonate thymocytes (83). In the same line, Silva-Santos and colleagues observed a reduction in the frequency of IL-17A-producing Vγ6^+^ subset using double-heterozygous mice for the CD3 subunits γ and δ in which TCR signaling is attenuated (81).

These apparently contradictory results may have multiple explanations. Notably, it is assumable that the different subsets of γδT17 precursors may require a specific and fine-tuned TCR signals to engage in their differentiation program. Specifically, Vγ6^+^ may require an “intermediate” TCR signals while Vγ4^+^ subsets may need weak or no signals. Moreover, intensity of TCR signaling can be hardly compared from one experimental setting to another, making any generalization risky. In this context, the importance of TCR signaling in programming γδT17 cell differentiation is still an open question. This also raises the putative existence of TCR self-ligands for natural γδT17 cells. The recent discovery of butyrophilin-like molecules as Ags for mouse γδT cells (84, 85) opens a new exciting avenue of research in the field. Identification of the enlarged butyrophilin family in both mouse and human γδT cell biology might offer an interesting anchoring point for future translational studies.

#### *Costimulatory* *Molecules*

On top of the TCR, its accessory receptors have been proposed to participate in γδT cell differentiation. In addition to be a convenient marker to distinguish γδT functional subsets, the costimulatory receptor CD27 has been shown to participate in γδT cell development (14). Indeed, γδ thymocytes from *Cd27^−/−^* mice presented altered expression of *ifng*. Although CD27 deficiency did not influence the pool of γδT17 cells, CD27 gain of function in thymic cultures resulted in lower IL-17 transcripts by CD27^−^ γδ thymocytes (14). Thus, CD27 appears as a thymic regulator in γδT cell effector fate.

Inducible T cell co-stimulator (ICOS) signaling pathway has also recently emerged as a possible determinant in γδT17 cell (at least for the Vγ4^+^ subset) development (86). Agonistic activity of anti-ICOS mAb in fetal thymic organ culture significantly impaired Vγ4^+^ γδT17 development. In line, genetic ablation of ICOS tends to increase the pool of thymic Vγ4^+^ γδT17 cells (86). Thus, this study indicates that ICOS-dependent intracellular pathways in thymocytes controls γδT17 cell effector fate.

Besides, one must keep in mind, that, alongside with TCR and costimulation receptor signaling, multiple other signals have to be integrated by embryonic thymocytes to, *in fine*, engage toward the γδT17 effector fate.

#### *Soluble* *Mediator Receptor Signaling Pathways*

γδT17 precursors express specific receptors for various soluble factors produced in the thymic environment by hematopoietic and non-hematopoietic cells. Signals provided by these mediators have to be integrated by γδT cells to fully develop.

For instance, TGFβ receptor (TGFβR) signaling pathway in developing γδT17 cells could be important in their effector fate. Mice deficient for Smad3, a critical component of the TGFβR signaling pathway presented a striking defect in frequency of thymic γδT17 cells compared with littermate controls (71). However, since the authors did not provide direct evidence (bone marrow chimera and OP-9 models) for an intrinsic role of the TGFβR signaling pathway, it cannot be excluded that this pathway is indirectly linked to γδT17 cell development.

In addition, the targeting of lymphotoxin-β receptor through double positive thymocytes-derived ligands (87) also controls the generation of γδT17 cells by regulating the expression of TFs from the NF-κB family namely RelA and RelB (88).

As mentioned above, the IL-7/IL-7Rα axis is important to generate a normal pool of γδT17 cells. However, a better understanding of the downstream molecular cascade involved will be helpful to understand whether this axis controls the differentiation program or the homeostasis of γδT17 cells.

On the other hand, IL-15Rα signaling disruption favors the development of γδT17 cells in thymus of neonates (65). The molecular mechanisms responsible for this are currently unknown but might rely on the activation of repressing factors in the IL-15Rα signaling pathway or could be indirect by reducing the competition with γδT17-driving γ-chain-dependent cytokines such as IL-7.

The expression of the prostacyclin (PGI2) receptor (IP) on thymocytes has also been demonstrated to control γδT17 cell development. This was evidenced by a failure to generate γδT17 cells in the thymus of IP^−/−^ mice (89). However, the molecular determinants involved in this process are yet to be defined.

#### The γδT17 Cell Transcriptional Program

**Figure 2:**
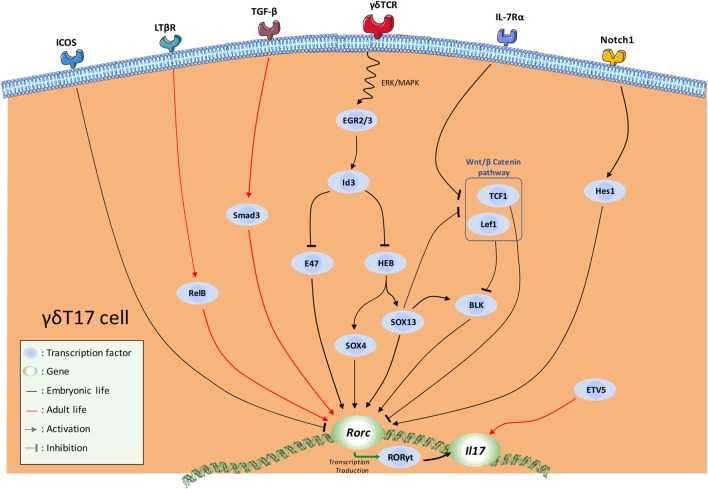
Schematic overview of the current knowledge in the transcription factor (TF) network involved in natural γδT17 cell effector fate. Major activating and repressing pathways implicated during γδ17 effector fate acquisition are depicted.

The dynamic integration of these multiple signals leads to the implementation of a complex transcriptional program that dictates γδT17 cell effector fate (Figure 2). The aim of this program is ultimately to maintain and/or to favor the expression of Rorc (encoding RORγt), the cardinal TF for IL-17-secreting cells (90) including γδT cells (72). The recent advances in RNA deep sequencing analysis allowed a better understanding of the transcriptional regulation involved in γδT17 development.

Thus, SRY-related HMG-box (SOX) 4 and SOX13, two members of the high mobility group box TF family constitute a central node of regulation in this program (50, 91, 92). SOX4/13 are paramount in the acquisition of the γδT17 effector fate by (1) directly controlling *Rorc* transcription, (2) possibly enhancing important γδT17 cell-driving pathways such as IL-7Rα signaling, and (3) possibly inhibiting *Rorc*-repressing TFs (31, 92). In this later mechanism, SOX13 was suggested to inhibit the *Rorc*-repressing activity of two downstream mediators of the Wnt/β-catenin signaling pathway namely lymphoid enhancer-binding factor 1 (Lef1) and transcription factor 1 (TCF1) (92). In this context, *Tcf7* (encoding for TCF1) deficiency leads to an aberrant high proportion of γδT17 cells (92). At this stage, it is also important to mention that this pathway also regulates the development of the IFN-γ-producing Vγ1Vδ6.3^+^ and Vγ5^+^ cells (92). The mechanisms by which the TCF1–Lef1 axis counteracts the γδT17 transcriptional program are not fully understood. First, it is possible that TCF1 and Lef1 control *Rorc* expression through epigenetic (histone deacetylase) activity as suggested in conventional T cells (93). Alternatively, this axis could also indirectly repress *Rorc* expression by inhibiting the transcription of B lymphocyte kinase (Blk) (92), an important signal transducer in γδT17 cell development (94). However, how Blk controls RORγt expression is currently unknown. It is noteworthy that, using *blk^−/−^* mice, Vγ6^+^ T cells were shown to be more Blk-dependent than Vγ4^+^ (94). As SOX13 was shown to regulate Blk expression and to control Vγ4^+^, but minimally Vγ6^+^, subset development (92), these results appear somewhat contradictory. However, Vγ4 and Vγ6 subsets develop at different temporal windows; therefore, they are likely to integrate different thymic signals. As a result, this might modulate the relative importance of a same regulatory axis, and eventually leading to different effects on their respective transcriptional program. Thus, regulatory network required for Vγ4^+^ ontogeny may be more SOX13-dependent than Vγ6^+^ subset. Regarding the differential contribution of the TCR signaling in the γδT17 effector fate of these populations, Blk may play a role at this stage. According to its regulatory activity on TCR signaling, Blk could act as a “rheostat” to fine-tune signals delivered by the thymic γδT cell ligands. Thus, Blk deficiency might affect more Vγ6^+^ ontogeny as TCR signaling has been proposed to control the development of these latter but not Vγ4^+^. Of note, Blk overexpression has been shown to enhance IL-7 responsiveness in B cells (95).

As stated earlier, the TCR signaling pathway strongly influences the acquisition of the γδT17 effector fate. Mechanistically, TCR engagement induces upregulation of proteins of the early growth response (Egr) family namely Egr2 and Egr3 (31, 81). These two TFs positively regulate the DNA-binding protein inhibitor Id3. Thus, Id3 impairs γδT17 cell differentiation through (1) inhibition of HeLa E-box binding protein (HEB)-dependent *Sox4* and *Sox13* expressions (96) and (2) inhibition of the *Rorc* promoter E47 (91). This scheme is also in line with the differential requirement for TCR strength in γδT17 cell effector fate of Vγ6^+^ and Vγ4^+^ further emphasizing the differences in the developmental programs of Vγ6^+^ *vs* Vγ4^+^ γδT17 cells.

In addition, the promyelocytic leukemia zinc finger (PLZF) protein is a key TF in the development of some innate and innate-like lymphocytes that dictates their acquisition of a Th-like effector program (97–99). PLZF was shown to control Vγ6^+^ differentiation into γδT17 cells (100). The molecular mechanisms that govern PLZF activity in Vγ6^+^ development are currently unknown and require further investigations. PLZF contribution in Vγ4^+^ γδT17 cell development has not been assessed yet. However, it is noteworthy that PLZF does not appear to be expressed in neonates Vγ4^+^ precluding a role of this TF for this particular subset (100).

Among the intracellular pathways that dictate the γδT17 effector fate, Notch signaling contributes to the generation of γδT17 cells through the helix-loop-helix protein Hes1 (78). Since it mainly exerts transcriptional repressing activities, it is possible that Hes1 acts in one of the pathways discussed above. Unrevealing the Hes1 interactome in developing γδT17 cells will be informative to get insight into the molecular factors that regulate this mechanism. An additional pathway by which the Notch signaling pathway could influence γδT17 cell development is by promoting IL-7Rα expression through the RBP-Jκ pathway (68). However, this pathway was only described in peripheral γδT17 cells of adult mice to control their homeostasis and self-renewal.

In addition to its role in proliferation/survival of γδT17 cells during development, the IL-7Rα signaling pathway is also likely to contribute to their transcriptional program. Indeed, IL-7/IL-7Rα signaling in fetal thymocytes was demonstrated to blunt both *Lef1* and *Tcf7* expression (101).

*In silico* analyses have also been fruitful to understand the transcriptional program of γδT17 cells. Using an algorithm that predicts important regulators across various lineages, the TF ETV5, along with SOX13 was proposed as a master regulator in Vγ4^+^ γδT17 cell differentiation (102). Conditional ablation of ETV5 in T cells confirmed the role of this TF in Vγ4^+^ γδT17 effector fate (102). Absence of ETV5 in developing Vγ4^+^ slightly reduced RORγt expression but severely impaired IL-17 secretion. This is reminiscent with the situation for Th17 cell differentiation in which ETV5 directly promotes *il17a* and *il17f* expressions but has no influence on *Rorc* (103). The role of ETV5 in Vγ6^+^ development remains to be determined; however, ETV5 is highly expressed on immature fetal Vγ6^+^ and strongly repressed upon maturation (http://www.immgen.org/databrowser/index.html).

Originally thought to be acquired by “neglect,” this literature underlines that the γδT17 effector fate is under the control of a very active process in which the SOX4/13 axis acts as a guardian for proper RORγt expression. In addition, the discrepancies in the phenotypes observed for Vγ4^+^ and Vγ6^+^ γδT17 cells imply different programs for natural γδT17 cell development. Thus, further transcriptomic analyses at single cell resolution are clearly required to better decipher the overlapping and/or specific developmental “trajectories” that drive the effector fate of these subsets.

### What Can We Learn From Transcriptomic Analysis of Developing Natural γδT17 Cells?

As stated above, the recent advances in the quality of whole genome analyses allowed to validate and/or to predict the involvement of numerous genes in the transcriptional signature of many cell populations including γδT cells. In addition, normalized and comparative analysis of various gene sets among lymphocyte lineages led to the identification of conserved and/or distinct signature pathways in their effector program including Th17(-like) effector fate (104).

**Figure 3:**
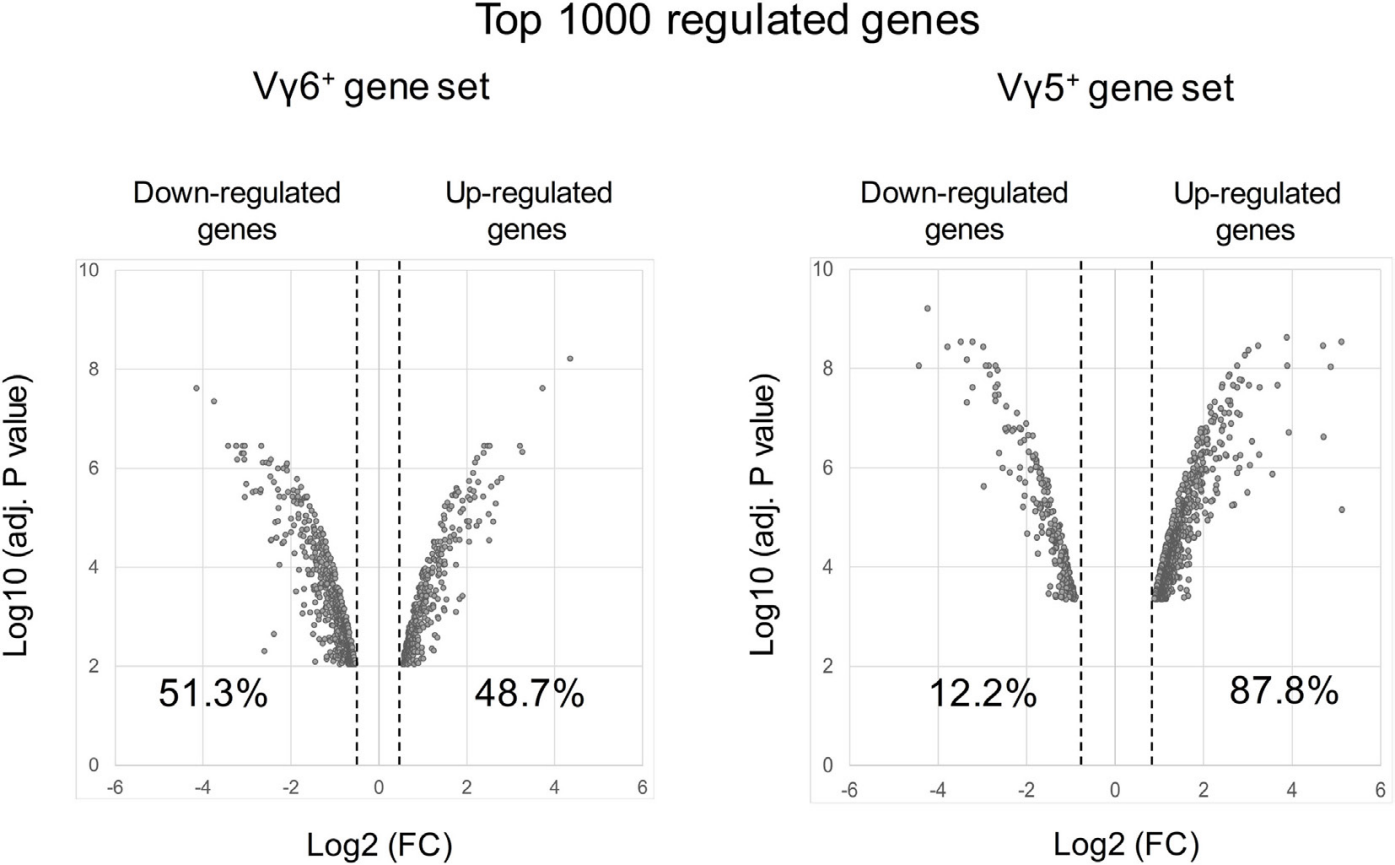
Volcano plots of the top 1,000 regulated genes during Vγ6 and Vγ5 maturation. Raw data were extracted from datasets (GSE37448 and GSE15907) downloaded from the NCBI’s data repositories. Vγ6^+^ and Vγ5^+^ gene sets were generated by comparing gene expression in immature (CD24^high^) vs mature (CD24^low^) thymocytes at E17 for both populations (2–3 replicates/subset). The top 1,000 regulated genes (*P* < 0.05) were used to constitute the two gene sets (Table S1 in Supplementary Material). Volcano plots represent either positively or negatively regulated genes (as fold change) according to their respective *P* value. Labels indicate the percentage of genes that are either positively or negatively regulated in each dataset. FC, fold change.

However, *in silico* analysis of the transcriptional program of γδT17 cells has been mainly discussed regarding the maturation of the Vγ4^+^ cell subset in adult mice (91, 92, 104). Thus, this population comprises both “inducible” γδT17 cells as well as non-IL-17-producing subsets. To focus on “natural” γδT17 cells that develop during embryonic life, we reanalyzed the datasets of developing Vγ6^+^ T cells (immature/CD24^hi^ vs mature/CD24^low^) (GSE37448). Bioinformatic analysis generated a gene set of the top 1,000 transcripts significantly regulated during the effector fate acquisition of this subset (Table S1 in Supplementary Material). Interestingly, while similar analysis on developing fetal Vγ5^+^ T cells (GSE15907) indicated that 87.8% of the 1,000 top regulated genes were up-regulated, only 48.7% did so in the Vγ6^+^ dataset (Figure 3). This emphasizes the fact that, unlike γδT1, γδT17 cell effector fate is rather acquired using a repressing model.

As expected, among the list of gene generated, we found many up-regulated genes shared with other innate(-like) or adaptive IL-17-producing lymphocytes including *Rorc, Il17a*, and *Il17f* and the cytokine/chemokine receptors *Il1r1, Il2rb, Il7r, Il17rc, Il17re, Il23r, Il18r1, Ccr6*, and *Cxcr6*. This is paralleled by a silencing of genes involved in Th1 and Th2 differentiation, such as *Il2ra, Il12rb2, Lck, Gata3*, and *Maml2*.

In addition, we noted numerous genes involved in TCR signaling including *Lck, Nck2, Pak1, Plcg, Prkcq, Ptpn22*, and *Nfkbie*. Notably, all these transcripts were down-modulated during Vγ6^+^ maturation. Moreover, genes involved in costimulation, such as *Cd27, Cd28, Icos, Themis, Slamf1, Slamf6*, and *Pik3r2*, were also repressed upon differentiation. In line, it is noteworthy that the transcript encoding for the nuclear receptor Nur77 (*Nr4a1*), a faithful marker of TCR strength (105) is strongly repressed during Vγ6^+^ maturation. Given the controversy discussed previously, these observations clearly suggest that the TCR signaling pathway has to be maintained under tight regulation to allow Vγ6^+^ T cell differentiation.

**Figure 4:**
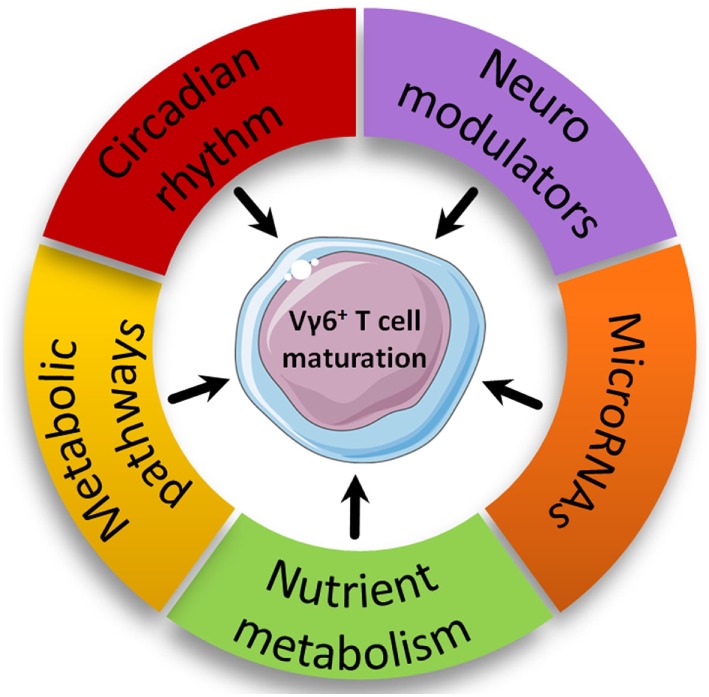
Proposed new biological pathways involved in intrathymic Vγ6^+^ maturation. Biological pathways with enriched modulated genes in the Vγ6^+^ dataset are represented based on advanced pathway analysis using the trial version of iPathwayGuide (©Advaita Corporation).

More importantly, using advanced pathway analysis, we pinpointed, in the Vγ6^+^ T cell gene set, multiple family of genes involved in biological processes and molecular pathways that might be involved in their developmental program (Figure 4).

#### Nutrient Metabolism

Along with *Nr4a1*, other genes encoding for nuclear receptors such as receptors of vitamin A (*Rarg*) and D (*Vdr*), two vitamins reported to participate in ILC, γδT and NKT (25, 106) homeostasis and development are up-regulated during Vγ6^+^ maturation. In addition, genes encoding for vitamin transporters (*Slc23a2* and *Slc2a3*) were also modulated in the Vγ6^+^ gene set. This could imply an important role for vitamins in γδT17 cell development. Somewhat related, expression of *gpr183*, a sensor of oxysterols was strongly up-regulated (3.6 fold) in developing Vγ6^+^ T cells. Interestingly, GPR183 has recently emerged as a critical player in the control of ILC3 homeostasis (107). Moreover, oxysterols are ligands for RORγt and drive Th17 cell differentiation (108). The influence of nutrient-derived metabolites on lymphocyte immunity including early development has recently gained considerable attention (2). The recent discovery of vitamin B2 metabolites as Ags for MAIT cells (109) will certainly reinforce the interest of immunologists for nutrient metabolism. The availability of vitamins *in utero* has also been shown to control the quality of the immune system in later life (110). According to these arguments and the temporal window of development for γδT17 cells, investigating the nutrient metabolism in γδT17 cell biology will be likely to provide new interesting data on the influence of maternal diet in shaping immunity.

#### Neuroimmunology

In line with the emerging concept of neuroimmunology, numerous members of the “neuroactive ligand-receptor interaction” pathway were present in the Vγ6^+^ gene set including genes encoding for neuroactive substance receptors of neuropeptide (*Gpr83*), hormones (*Sstr2, Rxfp1*, and *Calcrl*), prostanoids (*Ptger4, Ptgfrn*, and *Ptgir*), leukotrienes (*Cysltr2* and *Ltb4r1*), nucleotides (*P2rx7, Adora2a*, and *Lpar4*), and amino acids (*Gabbr1, Gabbr2*, and *Gria3*). Interestingly, the above-mentioned genes encoding for prostanoids, hormones, and nucleotide receptors are down-regulated during Vγ6^+^ T cell maturation, while those encoding for leukotrienes are up-regulated.

The relationship between neuromodulators and immune cell development is largely unexplored. However, there are evidence for an expression of neuromodulators and their associated-receptors in TEC and on thymocytes, respectively (111). Furthermore, *ex vivo* somatostatin (ligand for *Sstr2*) addition in FTOC increased thymocyte numbers and maturation. By contrast, both neuropeptide Y (ligand for *Gpr83*) and calcitonin (ligand for *Calcrl*) reduced thymocyte numbers (112). Last, *Ptgir* (encoding for the prostaglandin I2 receptor) has already been biologically validated to participate in natural γδT17 cell development (89). Regarding this, a broad analysis of the eicosanoid family in γδT17 cell development should be encouraged.

#### Circadian Rhythm

Among highly regulated genes, our analysis also retrieved genes related to circadian rhythm. Thus, we found that *Nr1d1, Nr1d2*, and *Bhlhe40*, which encodes for REV-ERBα, REV-ERBβ, and Dec1 proteins, respectively, were all up-regulated in mature Vγ6^+^ T cells. These proteins are critical repressors of *Arntl* (encoding for Bmal1), *Npas2*, and *Clock*, three master clock genes (113, 114). Of note, *Arntl, Npas2*, and *Clock* were also significantly regulated in developing Vγ6^+^ T cells. Recent literature has emphasized the importance of circadian rhythms in fine-tuning immune responses (113, 115). Interestingly, RORγt expression has been shown to be under circadian regulation through a REV-ERBα-dependent mechanism (116). Thus, these data may suggest a role for the circadian rhythm in Vγ6^+^ T cell biology. Of note, NFIL3 (E4BP4), a repressor of the circadian clock, is implicated in the differentiation/development of ILCs and NK cells (117–119). In addition, we can speculate that the regulation of these clock genes helps mature Vγ6^+^ T cells to integrate and to regulate circadian cues once in the periphery, as demonstrated for many other cellular actors of innate immunity (113).

#### Immunometabolism

Regarding the growing interest for understanding immunometabolic pathways implicated in leukocyte biology, we searched for genes involved in metabolic pathways. Our analysis revealed that many genes involved in the six major metabolic pathways specifically glycolysis (*Aldh2, Ldhb, Acss1*, and *Acss2*), tricarboxylic acid cycle (*Idh1, Idh2*, and *Aco1*), pentose phosphate pathway (*Fbp1*), fatty acid oxidation (*Cpt1a, Nr4a3, Peci, Auh, Pex5*, and *Ivd*), fatty acid synthesis (*Fads2* and *Slc45a3*), and amino acid metabolism (*Aco1* and *Bcat1*) are down-regulated in mature Vγ6^+^ T cells.

Interestingly, fatty acid oxidation is associated with regulatory T cell differentiation while glycolysis is a major metabolic pathway in effector T cell differentiation (120, 121). Of note, expression of *Cpt1a* was reduced in Th17 cells compared with regulatory T cells (120). Enhanced fatty acid synthesis and glycolysis in immune cells, especially T cells, have been regarded as markers of inflammatory cells required for acquisition of effector functions upon inflammatory conditions (122). This adds a metabolic argument into the fact that Vγ6^+^ T cells are “preset” cells with metabolic programing occurring during development, to be immediately and fully functional once in peripheral tissues.

#### MicroRNAs

To date, there is limited literature on the role of microRNAs in leukocyte development. In our gene set, we detected the presence of five microRNAs (*mir15b, mir181a-1, mir181a-2, mir181b-1*, and *mir181b-2*), all of them being down-regulated in mature Vγ6^+^ T cells. Of note, miR-181 was reported to be essential in NKT cell development (123, 124). Interestingly, the group of Immo Prinz studied the impact of miR-181a/b-1 deficiency on γδT cell development. While thymic Vγ1^+^ and Vγ4^+^ T cell subsets were unaltered in the absence of miR-181a/b-1, authors reported a higher frequency of thymic, but not peripheral Vγ6^+^ cells in miR-181a/b-1-deficient mice (125). The reason that underlines this feature is currently unknown. However, since miR-181 is a well-known positive regulator of the TCR signal strength (126), it is tempting to hypothesize that a reduced TCR signaling confers an advantage for Vγ6^+^ T cell differentiation. Thus, defining the miRome of developing γδT17 cells will potentially bring a novel layer of complexity in their developmental program.

Even if these *in silico* analyses suggest a role for scantily explored biological pathways in γδT17 cell development and/or maintenance, supportive experimental data are clearly required to explore these predictive hypotheses. It is noteworthy that these proposed biological pathways are not meant to be Vγ6^+^ cell-specific and, therefore, it does not preclude that other cell types including other γδT cell subsets may rely on similar biological pathways to develop. In addition, instruction that drives γδT17 effector fate is likely to start early in thymocyte development [even before TCR rearrangement (49)]; therefore, comparing immature vs mature populations probably induced an important bias in our analysis.

## Concluding Remarks

Despite the considerable body of work performed in the field of γδT17 cell ontogeny, many questions remain unsolved and sometimes appear more complex than initially thought. Even if revealing bulk transcriptomes have been informative to predict the developmental program of γδT17 cells, they present a major drawback since this kind of analysis does not reflect the dynamic aspect of the development. In this context, the recent and rapid evolution in single cell deep RNA sequencing and bioinformatics technologies will undoubtedly help to reveal the developmental “trajectories” that dictate γδT17 cell effector fate. In addition, other layers of regulation such as post-transcriptional (especially epigenetic) regulation and implication of microRNAs deserve further investigations and will have to be integrated in order to better decipher the general mechanism(s) driving γδT17 cell development. Given the critical role of γδT17 cells in major health concerns such as infections and cancer, advances in these fundamental biological processes are clearly mandatory.

## Author Contributions

YJ, EP, MH, MS-T, TB, and CP prepared and wrote the manuscript.

## Conflict of Interest Statement

The authors declare that the research was conducted in the absence of any commercial or financial relationships that could be construed as a potential conflict of interest.

## Abbreviations

Ag, antigen; APC, Ag presenting cells; Blk, B lymphocyte kinase; DETC, dendritic epidermal γδT cell; IL, interleukin; IFN, interferon; ILC, innate lymphoid cells; Lef1, lymphoid enhancer-binding factor; MAIT, mucosal-associated invariant T; NKT, Natural Killer T; TCR, T cell receptor; TEC, thymic epithelial cells; TF, transcription factor.
