# Supplementary material for: Thymic Program Directing the Functional Development of γδT17 Cells
Source: Front Immunol. 2018 May 8;9:981. doi: 10.3389/fimmu.2018.00981 (PMC5951931; doi:10.3389/fimmu.2018.00981)
Supplement: Supplementary file 1 [file Table_1.PDF]

Table S1: Top 1000 regulated genes during fetal Vγ6+ T cell maturation

Gene symbol	Gene description	Fold change	P value
Il23r	interleukin 23 receptor	**20,449**	6,068E-09
Clnk	cytokine-dependent hematopoietic cell linker	**13,241**	2,393E-08
Serpinb1a	serine (or cysteine) peptidase inhibitor clade B member 1a	**9,636**	4,687E-07
Il18r1	interleukin 18 receptor 1	**9,279**	3,526E-07
Itgb5	integrin beta 5	**6,906**	1,564E-06
Klrb1f	killer cell lectin-like receptor subfamily B member 1F	**6,441**	1,880E-06
Tns4	tensin 4	**6,319**	5,211E-06
Sparc	secreted acidic cysteine rich glycoprotein	**6,056**	1,201E-05
Man1c1	mannosidase alpha class 1C member 1	**5,890**	2,340E-06
Abi3bp	ABI gene family member 3 (NESH) binding protein	**5,752**	3,526E-07
Il1r1	interleukin 1 receptor type I	**5,730**	2,839E-05
Klra5	killer cell lectin-like receptor subfamily A member 5	**5,637**	8,441E-06
Gpr44	G protein-coupled receptor 44	**5,559**	3,526E-07
Myo1e	myosin IE	**5,276**	3,526E-07
Fgl2	fibrinogen-like protein 2	**5,214**	4,860E-07
Npl	N-acetylneuraminate pyruvate lyase	**5,190**	3,681E-06
Il17a	interleukin 17A	**4,862**	1,174E-05
Slc15a3	solute carrier family 15 member 3	**4,764**	1,880E-06
Cdh10	cadherin 10	**4,689**	6,139E-07
Zp1	zona pellucida glycoprotein 1	**4,650**	1,433E-05
Adam8	a disintegrin and metallopeptidase domain 8	**4,560**	7,597E-07
Srgap3	SLIT-ROBO Rho GTPase activating protein 3	**4,530**	3,832E-06
Sepp1	selenoprotein P plasma 1	**4,438**	2,880E-06
Cpd	carboxypeptidase D	**4,418**	1,228E-06
Hbegf	heparin-binding EGF-like growth factor	**4,222**	2,660E-06
St3gal6	ST3 beta-galactoside alpha-23-sialyltransferase 6	**4,197**	4,314E-06
Raver2	ribonucleoprotein PTB-binding 2	**4,155**	3,016E-05
LOC100504914		**4,140**	1,805E-06
Srgap3	SLIT-ROBO Rho GTPase activating protein 3	**4,134**	1,174E-05
Nr1d1	nuclear receptor subfamily 1 group D member 1	**4,114**	1,500E-05
Naga	N-acetyl galactosaminidase alpha	**4,087**	3,795E-06
Npas2	neuronal PAS domain protein 2	**4,002**	1,174E-05
Fah	fumarylacetoacetate hydrolase	**3,779**	6,640E-06
Cxcr6	chemokine (C-X-C motif) receptor 6	**3,744**	3,758E-04
Plxnc1	plexin C1	**3,734**	4,458E-06
Gpr183	G protein-coupled receptor 183	**3,623**	7,709E-06
Il18rap	interleukin 18 receptor accessory protein	**3,574**	1,724E-05
Vdr	vitamin D receptor	**3,570**	4,309E-04
Mtap1b	microtubule-associated protein 1B	**3,557**	2,735E-05
Acpp	acid phosphatase prostate	**3,500**	3,008E-06
Rnf43	ring finger protein 43	**3,423**	2,660E-06
Dock5	dedicator of cytokinesis 5	**3,391**	2,528E-06
F2rl2	coagulation factor II (thrombin) receptor-like 2	**3,374**	3,526E-06
Capg	capping protein (actin filament) gelsolin-like	**3,373**	8,831E-05
Rbpms2	RNA binding protein with multiple splicing 2	**3,366**	7,899E-04
Zdhhc2	zinc finger DHHC domain containing 2	**3,353**	2,735E-05

Sept8	septin 8	3,331	3,433E-06
Adam12	a disintegrin and metallopeptidase domain 12 (meltrin alpha)	3,318	3,339E-06
Stac2	SH3 and cysteine rich domain 2	3,273	2,182E-05
Dlg5	discs large homolog 5 (Drosophila)	3,198	1,567E-05
Matn2	matrilin 2	3,196	3,470E-06
Ccr6	chemokine (C-C motif) receptor 6	3,150	5,769E-06
Chad	chondroadherin	3,096	2,384E-05
Il17f	interleukin 17F	3,072	4,078E-05
Susd2	sushi domain containing 2	3,043	6,640E-06
Osbpl3	oxysterol binding protein-like 3	3,028	3,025E-05
Gcnt1	glucosaminyl (N-acetyl) transferase 1 core 2	2,947	4,964E-06
Car13	carbonic anhydrase 13	2,911	4,403E-04
Aqp3	aquaporin 3	2,842	5,769E-06
Cd44	CD44 antigen	2,835	3,871E-05
Scpep1	serine carboxypeptidase 1	2,834	2,735E-05
Sntb1	syntrophin basic 1	2,826	8,741E-05
Bcl2a1a	B-cell leukemia/lymphoma 2 related protein A1a	2,815	7,572E-05
St6galnac3	ST6 (alpha-N-acetyl-neuraminyl-23-beta-galactosyl-13)-N-acetylgalactosaminide alpha-26-sialyltransferase 3	2,807	7,374E-04
Slc27a6	solute carrier family 27 (fatty acid transporter) member 6	2,805	1,336E-04
Socs2	suppressor of cytokine signaling 2	2,804	1,724E-05
Pde5a	phosphodiesterase 5A cGMP-specific	2,799	1,041E-05
Igsf11	immunoglobulin superfamily member 11	2,793	8,831E-05
S100a6	S100 calcium binding protein A6 (calcyclin)	2,784	6,745E-04
Atp2b4	ATPase Ca++ transporting plasma membrane 4	2,783	8,783E-06
Plxnd1	plexin D1	2,753	2,577E-04
F2r	coagulation factor II (thrombin) receptor	2,722	4,309E-05
Serinc2	serine incorporator 2	2,714	1,391E-05
Kcnk1	potassium channel subfamily K member 1	2,696	4,063E-05
Furin	furin (paired basic amino acid cleaving enzyme)	2,676	2,002E-04
Id2	inhibitor of DNA binding 2	2,672	5,475E-05
Jag1	jagged 1	2,668	7,631E-05
Il17re	interleukin 17 receptor E	2,660	5,475E-05
Art2a-ps	ADP-ribosyltransferase 2a	2,653	1,304E-05
Crybg3	beta-gamma crystallin domain containing 3	2,631	3,025E-05
Ptplad2	protein tyrosine phosphatase-like A domain containing 2	2,606	4,078E-05
Fam124b	family with sequence similarity 124 member B	2,592	1,361E-04
Acot9	acyl-CoA thioesterase 9	2,584	5,475E-05
Bcl2a1d	B-cell leukemia/lymphoma 2 related protein A1d	2,581	7,076E-05
Itsn1	intersectin 1 (SH3 domain protein 1A)	2,572	1,122E-04
Bcl2a1c	B-cell leukemia/lymphoma 2 related protein A1c	2,568	8,858E-05
Esrp1	epithelial splicing regulatory protein 1	2,561	1,076E-03
Bcl2a1b	B-cell leukemia/lymphoma 2 related protein A1b	2,554	7,195E-05
Lysmd2	LysM putative peptidoglycan-binding domain containing 2	2,544	1,452E-04
Mboat2	membrane bound O-acyltransferase domain containing 2	2,531	1,000E-03
Spon2	spondin 2 extracellular matrix protein	2,529	4,396E-04
1700012B07Rik	RIKEN cDNA 1700012B07 gene	2,527	4,934E-05
Nckap1	NCK-associated protein 1	2,526	3,025E-05
Klrd1	killer cell lectin-like receptor subfamily D member 1	2,509	2,653E-03
Glyat	glycine-N-acyltransferase	2,470	4,239E-05

Lrrc25	leucine rich repeat containing 25	2,450	2,893E-04
Myo1f	myosin IF	2,437	7,191E-05
2410066E13Rik	RIKEN cDNA 2410066E13 gene	2,420	2,351E-03
Chst11	carbohydrate sulfotransferase 11	2,419	3,100E-05
Rhobtb1	Rho-related BTB domain containing 1	2,401	3,871E-05
Rasa4	RAS p21 protein activator 4	2,393	3,758E-04
Ly6g5b	lymphocyte antigen 6 complex locus G5B	2,387	4,815E-03
Tasp1	taspase threonine aspartase 1	2,386	6,775E-05
Spock2	sparc/osteonectin cwcv and kazal-like domains proteoglycan 2	2,385	3,025E-05
Coro2b	coronin actin binding protein 2B	2,362	6,780E-05
AB041803	cDNA sequence AB041803	2,355	6,461E-04
Cpeb2	cytoplasmic polyadenylation element binding protein 2	2,353	5,868E-04
Crmp1	collapsin response mediator protein 1	2,345	1,021E-04
Tnfrsf25	tumor necrosis factor receptor superfamily member 25	2,343	5,799E-04
Ltb4r1	leukotriene B4 receptor 1	2,321	5,275E-05
Fam185a	expressed sequence AI847670	2,316	1,253E-04
Mmp25	matrix metallopeptidase 25	2,311	1,191E-04
Il20ra	interleukin 20 receptor alpha	2,301	4,368E-03
Rnase4	angiogenin ribonuclease RNase A family 5	2,291	1,328E-04
Fstl4	follistatin-like 4	2,284	3,544E-04
Edil3	EGF-like repeats and discoidin I-like domains 3	2,284	6,952E-04
Tnf	tumor necrosis factor	2,275	1,300E-03
March3	membrane-associated ring finger (C3HC4) 3	2,254	2,457E-04
B4galnt4	beta-14-N-acetyl-galactosaminyl transferase 4	2,227	7,297E-05
Trdn	triadin	2,209	6,745E-04
Acot10	acyl-CoA thioesterase 10	2,192	3,016E-04
Snx2	sorting nexin 2	2,172	1,135E-04
Golm1	golgi membrane protein 1	2,168	1,452E-04
Sec11c	SEC11 homolog C (S. cerevisiae)	2,163	1,169E-03
Tmem64	transmembrane protein 64	2,162	1,689E-04
Abca3	ATP-binding cassette sub-family A (ABC1) member 3	2,160	6,994E-05
Grifin	galectin-related inter-fiber protein	2,156	1,429E-03
Nav2	neuron navigator 2	2,145	2,656E-04
Sgms1	sphingomyelin synthase 1	2,120	1,739E-04
Cd160	CD160 antigen	2,108	7,910E-04
Farp1	FERM RhoGEF (Arhgef) and pleckstrin domain protein 1 (chondrocyte-derived)	2,105	3,797E-04
Stac	src homology three (SH3) and cysteine rich domain	2,104	1,371E-04
Il2rb	interleukin 2 receptor beta chain	2,102	3,991E-04
Ccdc90a	coiled-coil domain containing 90A	2,094	1,543E-04
Smox	spermine oxidase	2,094	5,025E-04
Slc35f2	solute carrier family 35 member F2	2,091	4,110E-04
Arrb1	arrestin beta 1	2,087	2,159E-04
Map4k5	mitogen-activated protein kinase kinase kinase kinase 5	2,085	4,530E-04
Sema6a	sema domain transmembrane domain (TM) and cytoplasmic domain (semaphorin) 6A	2,074	4,127E-04
Appl2	adaptor protein phosphotyrosine interaction PH domain and leucine zipper containing 2	2,073	2,199E-04
Irf5	interferon regulatory factor 5	2,073	2,640E-04
H2-K1	histocompatibility 2 K1 K region	2,072	1,231E-04
Samhd1	SAM domain and HD domain 1	2,069	1,051E-04

Osbpl6	oxysterol binding protein-like 6	2,064	3,018E-04
Gpr114	G protein-coupled receptor 114	2,061	3,239E-04
Zfp36l1	zinc finger protein 36 C3H type-like 1	2,057	7,531E-04
Lax1	lymphocyte transmembrane adaptor 1	2,056	1,049E-03
H2-gs10	MHC class I like protein GS10	2,054	1,087E-04
B930095G15Rik	RIKEN cDNA B930095G15 gene	2,046	5,401E-04
Gabbr1	gamma-aminobutyric acid (GABA) B receptor 1	2,042	4,762E-04
Ikzf3	IKAROS family zinc finger 3	2,039	6,445E-04
Mitf	microphthalmia-associated transcription factor	2,038	1,510E-04
Rbl2	retinoblastoma-like 2	2,032	7,647E-04
Slc23a2	solute carrier family 23 (nucleobase transporters) member 2	2,027	1,123E-04
Tacstd2	tumor-associated calcium signal transducer 2	2,019	1,635E-03
Mei4	RIKEN cDNA 4930486G11 gene	2,012	7,060E-03
Cnrip1	cannabinoid receptor interacting protein 1	2,011	5,799E-04
Rnf128	ring finger protein 128	2,009	6,751E-04
Socs2	suppressor of cytokine signaling 2	1,996	5,787E-04
Pfkp	phosphofructokinase platelet	1,995	1,630E-04
Nhs	Nance-Horan syndrome (human)	1,995	5,770E-04
Mtap7	microtubule-associated protein 7	1,993	1,221E-04
Cysltr2	cysteinyl leukotriene receptor 2	1,988	2,597E-04
Lgr4	leucine-rich repeat-containing G protein-coupled receptor 4	1,984	3,735E-04
Il17rc	interleukin 17 receptor C	1,977	1,361E-04
Pim2	proviral integration site 2	1,972	1,403E-03
Myadm	myeloid-associated differentiation marker	1,971	4,138E-04
Pigl	phosphatidylinositol glycan anchor biosynthesis class L	1,968	1,386E-04
Lfng	LFNG O-fucosylpeptide 3-beta-N-acetylglucosaminyltransferase	1,967	5,293E-03
Nxnl2	nucleoredoxin-like 2	1,967	6,816E-04
Rnf17	ring finger protein 17	1,961	4,217E-03
Dtx1	deltex 1 homolog (Drosophila)	1,954	6,937E-03
Ero1l	ERO1-like (S. cerevisiae)	1,953	6,745E-04
Nqo2	NAD(P)H dehydrogenase quinone 2	1,952	2,228E-04
Plekha8	pleckstrin homology domain containing family A (phosphoinositide binding specific) member 8	1,951	3,807E-04
Rorc	RAR-related orphan receptor gamma	1,941	7,427E-04
Fes	feline sarcoma oncogene	1,938	3,103E-04
Stx11	syntaxin 11	1,917	1,284E-03
Arnt2	aryl hydrocarbon receptor nuclear translocator 2	1,916	8,778E-04
Cish	cytokine inducible SH2-containing protein	1,915	3,606E-04
Marveld1	MARVEL (membrane-associating) domain containing 1	1,914	2,841E-04
Ell2	elongation factor RNA polymerase II 2	1,908	5,330E-04
Ahnak	AHNAK nucleoprotein (desmoyokin)	1,907	2,653E-04
Ttc39b	tetratricopeptide repeat domain 39B	1,905	1,889E-03
Mical2	microtubule associated monoxygenase calponin and LIM domain containing 2	1,904	3,221E-04
Sema4b	sema domain immunoglobulin domain (Ig) transmembrane domain (TM) and short cytoplasmic domain (semaphorin) 4B	1,904	3,239E-04
Gm4980	predicted gene 4980	1,896	3,758E-04
Bhlhe40	basic helix-loop-helix family member e40	1,877	9,499E-04
Ifi30	interferon gamma inducible protein 30	1,871	1,390E-03
Gramd1b	GRAM domain containing 1B	1,871	2,626E-04

Dusp7	dual specificity phosphatase 7	1,868	1,522E-03
Atxn1	ataxin 1	1,865	1,889E-03
Cd7	CD7 antigen	1,864	2,965E-03
Rnh1	ribonuclease/angiogenin inhibitor 1	1,863	7,101E-04
H2-D1	histocompatibility 2 D region locus 1	1,859	1,689E-03
Fam109b	family with sequence similarity 109 member B	1,854	8,566E-03
Irak3	interleukin-1 receptor-associated kinase 3	1,853	1,689E-03
Gm10786	predicted gene 10786	1,852	1,689E-03
Rasgef1b	RasGEF domain family member 1B	1,842	2,591E-03
Fxyd5	FXYD domain-containing ion transport regulator 5	1,842	8,801E-04
Arhgef3	Rho guanine nucleotide exchange factor (GEF) 3	1,840	7,455E-04
Trdn	triadin	1,838	5,135E-03
Prelid2	PRELI domain containing 2	1,837	3,779E-03
Tmem176a	transmembrane protein 176A	1,837	3,918E-03
Cpne8	copine VIII	1,836	6,461E-04
Tmem154	transmembrane protein 154	1,829	3,455E-03
Slc35d2	solute carrier family 35 member D2	1,825	2,613E-03
Lats2	large tumor suppressor 2	1,823	4,010E-04
Lrrn4	leucine rich repeat neuronal 4	1,822	7,668E-03
Nlrc5	NLR family CARD domain containing 5	1,816	7,647E-04
Tmem55a	transmembrane protein 55A	1,814	8,161E-03
Itga5	integrin alpha 5 (fibronectin receptor alpha)	1,811	1,887E-03
Dsg2	desmoglein 2	1,810	1,697E-03
Prkx	protein kinase X-linked	1,808	5,489E-04
Tec	tec protein tyrosine kinase	1,799	1,238E-03
Slco3a1	solute carrier organic anion transporter family member 3a1	1,798	1,183E-03
Flt3l	FMS-like tyrosine kinase 3 ligand	1,798	7,910E-04
Fam46a	family with sequence similarity 46 member A	1,796	8,070E-03
Atoh8	atonal homolog 8 (Drosophila)	1,794	1,363E-03
Nek6	NIMA (never in mitosis gene a)-related expressed kinase 6	1,793	7,157E-04
Dse	dermatan sulfate epimerase	1,793	2,800E-03
Lass4	LAG1 homolog ceramide synthase 4	1,790	1,007E-03
Atp2b1	ATPase Ca++ transporting plasma membrane 1	1,790	2,110E-03
H2-Q7	histocompatibility 2 Q region locus 7	1,789	1,443E-03
Tshz3	teashirt zinc finger family member 3	1,788	5,970E-04
Lta	lymphotoxin A	1,788	2,578E-03
Lmo4	LIM domain only 4	1,787	7,330E-04
Gprin3	GPRIN family member 3	1,781	8,801E-04
Nlrc5	NLR family CARD domain containing 5	1,775	1,106E-03
Wdr44	WD repeat domain 44	1,771	1,704E-03
Smap2	stromal membrane-associated GTPase-activating protein 2	1,767	6,909E-04
Amica1	adhesion molecule interacts with CXADR antigen 1	1,764	5,060E-03
Card10	caspase recruitment domain family member 10	1,764	2,823E-03
Tube1	epsilon-tubulin 1	1,761	4,403E-03
Serpinb6a	serine (or cysteine) peptidase inhibitor clade B member 6a	1,759	3,359E-03
Aebp2	AE binding protein 2	1,757	8,088E-04
Fnip2	folliculin interacting protein 2	1,756	5,587E-04
Stc2	stanniocalcin 2	1,755	2,653E-03
Pim1	proviral integration site 1	1,750	2,198E-03
Tnfrsf21	tumor necrosis factor receptor superfamily member 21	1,743	5,951E-04

Sdc1	syndecan 1	1,742	1,000E-03
Maf	avian musculoaponeurotic fibrosarcoma (v-maf) AS42 oncogene homolog	1,741	1,044E-03
Xlr4b	X-linked lymphocyte-regulated 4B	1,740	8,986E-03
Rarg	retinoic acid receptor gamma	1,739	1,353E-03
Dhrs7	dehydrogenase/reductase (SDR family) member 7	1,736	2,326E-03
Kcnk6	potassium inwardly-rectifying channel subfamily K member 6	1,735	7,531E-04
Rexo2	REX2 RNA exonuclease 2 homolog (S. cerevisiae)	1,735	7,629E-04
Hs3st1	heparan sulfate (glucosamine) 3-O-sulfotransferase 1	1,730	2,198E-03
Klrc1	killer cell lectin-like receptor subfamily C member 1	1,726	5,135E-03
Il7r	interleukin 7 receptor	1,721	5,898E-03
Krt24	keratin 24	1,720	7,577E-03
Ptk2	PTK2 protein tyrosine kinase 2	1,715	7,531E-04
Irf6	interferon regulatory factor 6	1,707	4,599E-03
She	src homology 2 domain-containing transforming protein E	1,705	3,448E-03
Phactr2	phosphatase and actin regulator 2	1,701	2,403E-03
Eif4e3	eukaryotic translation initiation factor 4E member 3	1,700	1,240E-03
D1Ertd622e	DNA segment Chr 1 ERATO Doi 622 expressed	1,698	4,397E-03
Rnft1	ring finger protein transmembrane 1	1,698	3,645E-03
Rbms1	RNA binding motif single stranded interacting protein 1	1,696	2,800E-03
Pde6d	phosphodiesterase 6D cGMP-specific rod delta	1,696	4,218E-03
B3gnt5	UDP-GlcNAc:betaGal beta-13-N-acetylglucosaminyltransferase 5	1,689	1,789E-03
Ttc39b	tetratricopeptide repeat domain 39B	1,686	1,567E-03
Cap2	CAP adenylate cyclase-associated protein 2 (yeast)	1,686	3,329E-03
Igf2bp2	insulin-like growth factor 2 mRNA binding protein 2	1,685	3,329E-03
Arhgap25	Rho GTPase activating protein 25	1,681	1,375E-03
Maged1	melanoma antigen family D 1	1,680	5,622E-03
Plekha1	pleckstrin homology domain containing family A (phosphoinositide binding specific) member 1	1,679	3,924E-03
Dennd4c	DENN/MADD domain containing 4C	1,673	3,136E-03
Mdfic	MyoD family inhibitor domain containing	1,672	4,162E-03
Kdelc2	KDEL (Lys-Asp-Glu-Leu) containing 2	1,665	1,397E-03
Mina	myc induced nuclear antigen	1,662	1,515E-03
Smyd2	SET and MYND domain containing 2	1,662	1,329E-03
Pi4k2a	phosphatidylinositol 4-kinase type 2 alpha	1,659	5,237E-03
Cdon	cell adhesion molecule-related/down-regulated by oncogenes	1,659	1,984E-03
Tspan18	tetraspanin 18	1,659	5,248E-03
Tnfsf14	tumor necrosis factor (ligand) superfamily member 14	1,653	8,001E-03
Pik3ap1	phosphoinositide-3-kinase adaptor protein 1	1,651	3,266E-03
Plcb4	phospholipase C beta 4	1,647	2,955E-03
Fam102a	family with sequence similarity 102 member A	1,645	4,207E-03
Fkbp14	FK506 binding protein 14	1,644	5,227E-03
Stx1a	syntaxin 1A (brain)	1,641	4,756E-03
Ipcef1	interaction protein for cytohesin exchange factors 1	1,638	2,579E-03
Clock	circadian locomoter output cycles kaput	1,637	5,984E-03
Mapkapk3	mitogen-activated protein kinase-activated protein kinase 3	1,635	1,959E-03
E2f7	E2F transcription factor 7	1,634	2,059E-03
Pros1	protein S (alpha)	1,633	2,295E-03
Nlrc5	NLR family CARD domain containing 5	1,632	3,619E-03
Odc1	ornithine decarboxylase structural 1	1,631	8,551E-03

Nek7	NIMA (never in mitosis gene a)-related expressed kinase 7	1,629	1,722E-03
Nlrc5	NLR family CARD domain containing 5	1,627	5,729E-03
Serpina3f	serine (or cysteine) peptidase inhibitor clade A member 3F	1,626	7,667E-03
Iffo2	intermediate filament family orphan 2	1,621	1,843E-03
Plekho2	pleckstrin homology domain containing family O member 2	1,619	5,016E-03
St6galnac6	ST6 (alpha-N-acetyl-neuraminyl-23-beta-galactosyl-13)-N-acetylgalactosaminide alpha-26-sialyltransferase 6	1,618	2,607E-03
Mfsd10	major facilitator superfamily domain containing 10	1,618	1,600E-03
Nlrc5	NLR family CARD domain containing 5	1,616	1,848E-03
Il27ra	interleukin 27 receptor alpha	1,615	5,135E-03
Fap	fibroblast activation protein	1,613	6,283E-03
Cerk	ceramide kinase	1,611	3,942E-03
Pear1	platelet endothelial aggregation receptor 1	1,610	4,393E-03
Prr13	proline rich 13	1,610	2,780E-03
Lpcat4	lysophosphatidylcholine acyltransferase 4	1,606	5,518E-03
Rcor3	REST corepressor 3	1,605	3,787E-03
Nr1d2	nuclear receptor subfamily 1 group D member 2	1,604	1,952E-03
Kctd9	potassium channel tetramerisation domain containing 9	1,604	5,263E-03
Plcd1	phospholipase C delta 1	1,601	2,608E-03
Fryl	furry homolog-like (Drosophila)	1,601	1,942E-03
Mrpl47	mitochondrial ribosomal protein L47	1,601	3,629E-03
Akirin1	akirin 1	1,598	6,975E-03
Gm2382	predicted gene 2382	1,598	3,606E-03
Enpp1	ectonucleotide pyrophosphatase/phosphodiesterase 1	1,595	3,386E-03
Zbtb7b	zinc finger and BTB domain containing 7B	1,595	5,681E-03
Btbd19	RIKEN cDNA 9530048O09 gene	1,594	2,443E-03
Tab2	mitogen-activated protein kinase kinase kinase 7 interacting protein 2	1,594	3,789E-03
Syne2	synaptic nuclear envelope 2	1,593	5,196E-03
Nlrc5	NLR family CARD domain containing 5	1,592	2,751E-03
Ryk	receptor-like tyrosine kinase	1,591	2,111E-03
Slc22a15	solute carrier family 22 (organic anion/cation transporter) member 15	1,589	2,955E-03
Nt5dc3	5'-nucleotidase domain containing 3	1,588	3,509E-03
Nlrc5	NLR family CARD domain containing 5	1,587	6,936E-03
Plek	pleckstrin	1,585	5,096E-03
Yes1	Yamaguchi sarcoma viral (v-yes) oncogene homolog 1	1,582	4,217E-03
Aebp1	AE binding protein 1	1,581	2,801E-03
Akirin2	akirin 2	1,580	4,434E-03
Egfl6	EGF-like-domain multiple 6	1,580	3,314E-03
Ptpn4	protein tyrosine phosphatase non-receptor type 4	1,580	7,031E-03
Lpcat3	lysophosphatidylcholine acyltransferase 3	1,580	4,931E-03
Ccl17	chemokine (C-C motif) ligand 17	1,578	3,942E-03
Tmem144	transmembrane protein 144	1,575	9,102E-03
Sbf2	SET binding factor 2	1,572	5,016E-03
4631416L12Rik	RIKEN cDNA 4631416L12 gene	1,570	8,178E-03
Prkar2a	protein kinase cAMP dependent regulatory type II alpha	1,568	8,662E-03
Irak2	interleukin-1 receptor-associated kinase 2	1,568	5,581E-03
Slc17a5	solute carrier family 17 (anion/sugar transporter) member 5	1,567	6,404E-03
Snrpb2	U2 small nuclear ribonucleoprotein B	1,566	8,070E-03
Odc1	ornithine decarboxylase structural 1	1,565	5,432E-03

1110018J18Rik	RIKEN cDNA 1110018J18 gene	1,565	6,510E-03
Gabbr2	gamma-aminobutyric acid (GABA) B receptor 2	1,564	4,599E-03
Il12rb1	interleukin 12 receptor beta 1	1,563	7,113E-03
Gm527	predicted gene 527	1,562	7,465E-03
H2-t9	histocompatibility 2 Q region locus 2	1,560	5,293E-03
Cept1	choline/ethanolaminephosphotransferase 1	1,559	8,046E-03
Tekt2	tektin 2	1,558	5,708E-03
Slc16a6	solute carrier family 16 (monocarboxylic acid transporters) member 6	1,557	3,918E-03
Gm3988		1,555	3,339E-03
Zfp831	zinc finger protein 831	1,551	8,070E-03
1110067D22Rik	RIKEN cDNA 1110067D22 gene	1,550	8,259E-03
Arl11	ADP-ribosylation factor-like 11	1,546	5,794E-03
Dnajb13	DnaJ (Hsp40) related subfamily B member 13	1,545	7,510E-03
Sec24d	Sec24 related gene family member D (S. cerevisiae)	1,543	7,923E-03
Zadh2	zinc binding alcohol dehydrogenase domain containing 2	1,543	6,865E-03
Shank2	SH3/ankyrin domain gene 2	1,541	8,070E-03
B3gnt3	UDP-GlcNAc:betaGal beta-13-N-acetylglucosaminyltransferase 3	1,541	4,815E-03
Klf10	Kruppel-like factor 10	1,539	3,709E-03
Itgb7	integrin beta 7	1,539	3,615E-03
Endod1	endonuclease domain containing 1	1,538	4,094E-03
Plk3	polo-like kinase 3 (Drosophila)	1,534	4,931E-03
Arhgef11	Rho guanine nucleotide exchange factor (GEF) 11	1,530	4,041E-03
Shank2	SH3/ankyrin domain gene 2	1,528	7,414E-03
Ncoa1	nuclear receptor coactivator 1	1,526	5,361E-03
Ttyh2	tweety homolog 2 (Drosophila)	1,524	7,491E-03
Fam38a	family with sequence similarity 38 member A	1,522	8,574E-03
Arntl	aryl hydrocarbon receptor nuclear translocator-like	1,522	5,074E-03
Nlrc5	NLR family CARD domain containing 5	1,518	8,103E-03
6030498E09Rik	RIKEN cDNA 6030498E09 gene	1,516	8,239E-03
Add3	adducin 3 (gamma)	1,513	4,918E-03
4930420K17Rik	RIKEN cDNA 4930420K17 gene	1,512	7,948E-03
Prrc1	proline-rich coiled-coil 1	1,507	5,043E-03
E330016A19Rik	RIKEN cDNA E330016A19 gene	1,504	7,010E-03
Igf2bp1	insulin-like growth factor 2 mRNA binding protein 1	1,504	8,281E-03
Abhd15	abhydrolase domain containing 15	1,503	8,195E-03
5330426P16Rik	RIKEN cDNA 5330426P16 gene	1,503	8,232E-03
Nudt4	nudix (nucleoside diphosphate linked moiety X)-type motif 4	1,501	7,380E-03
Slain2	SLAIN motif family member 2	1,496	5,258E-03
Abcc3	ATP-binding cassette sub-family C (CFTR/MRP) member 3	1,495	6,607E-03
Plp2	proteolipid protein 2	1,493	7,675E-03
Zbtb8a	zinc finger and BTB domain containing 8a	1,493	5,622E-03
Pkd1l3	polycystic kidney disease 1 like 3	1,492	6,120E-03
Faah	fatty acid amide hydrolase	1,491	5,843E-03
Olfr161	olfactory receptor 161	1,489	8,235E-03
Cbx7	chromobox homolog 7	1,488	9,120E-03

9930111J21Rik2	RIKEN cDNA 9930111J21 gene	1,486	7,675E-03
Fzd6	frizzled homolog 6 (Drosophila)	1,481	8,652E-03
Nlrc5	NLR family CARD domain containing 5	1,479	8,232E-03
Orai2	ORAI calcium release-activated calcium modulator 2	1,473	8,239E-03
Cited2	Cbp/p300-interacting transactivator with Glu/Asp-rich carboxy-terminal domain 2	1,463	8,911E-03
Nsf	N-ethylmaleimide sensitive fusion protein	1,461	9,115E-03
Anln	anillin actin binding protein	1,450	8,938E-03
Elovl5	ELOVL family member 5 elongation of long chain fatty acids (yeast)	-1,447	8,566E-03
Mns1	meiosis-specific nuclear structural protein 1	-1,447	8,980E-03
Gm17006	T cell receptor alpha variable 13-4/DV7	-1,458	7,577E-03
BC028528	cDNA sequence BC028528	-1,464	7,675E-03
Gnb1l	guanine nucleotide binding protein (G protein) beta polypeptide 1-like	-1,471	8,324E-03
Carkd	carbohydrate kinase domain containing	-1,473	8,040E-03
Lims1	LIM and senescent cell antigen-like domains 1	-1,479	7,541E-03
Pcyox1l	prenylcysteine oxidase 1 like	-1,479	7,702E-03
Gimap5	GTPase IMAP family member 5	-1,481	6,449E-03
Zmym3	zinc finger MYM-type 3	-1,486	6,502E-03
Mpa2l	macrophage activation 2 like	-1,486	6,533E-03
Fbxo32	F-box protein 32	-1,486	9,022E-03
Kbtbd11	kelch repeat and BTB (POZ) domain containing 11	-1,490	5,618E-03
Prkd2	protein kinase D2	-1,493	8,093E-03
Atp1b1	ATPase Na+/K+ transporting beta 1 polypeptide	-1,495	6,865E-03
Tgif1	TGFB-induced factor homeobox 1	-1,497	5,518E-03
Paip2	polyadenylate-binding protein-interacting protein 2	-1,497	6,449E-03
Sfxn4	sideroflexin 4	-1,502	7,386E-03
Igf1r	insulin-like growth factor I receptor	-1,504	8,000E-03
Rpl24	ribosomal protein L24	-1,504	5,168E-03
Rpl24	ribosomal protein L24	-1,504	5,984E-03
Stmn1	stathmin 1	-1,504	6,865E-03
Aco1	aconitase 1	-1,504	8,983E-03
Nt5c2	5'-nucleotidase cytosolic II	-1,506	6,676E-03
Nf1	neurofibromatosis 1	-1,508	5,545E-03
Tspan32	tetraspanin 32	-1,508	7,386E-03
Ikzf1	IKAROS family zinc finger 1	-1,508	6,417E-03
Rpl24	ribosomal protein L24	-1,511	5,295E-03
Hemgn	hemogen	-1,511	8,440E-03
Wdr31	WD repeat domain 31	-1,511	6,002E-03
Slc25a38	solute carrier family 25 member 38	-1,513	9,040E-03
Stmn1	stathmin 1	-1,513	5,600E-03
Ttc3	tetratricopeptide repeat domain 3	-1,515	7,984E-03
Sh3kbp1	SH3-domain kinase binding protein 1	-1,515	6,142E-03
Tmem66	transmembrane protein 66	-1,517	5,205E-03
3110007F17Rik	RIKEN cDNA 3110007F17 gene	-1,517	8,396E-03
Hbp1	high mobility group box transcription factor 1	-1,520	5,545E-03
Myl4	myosin light polypeptide 4	-1,520	6,912E-03
Mov10	Moloney leukemia virus 10	-1,522	5,212E-03
Egln3	EGL nine homolog 3 (C. elegans)	-1,522	6,097E-03

Tmx4	thioredoxin-related transmembrane protein 4	-1,524	6,828E-03
St3gal1	ST3 beta-galactoside alpha-23-sialyltransferase 1	-1,524	6,369E-03
Hsdl1	hydroxysteroid dehydrogenase like 1	-1,524	8,911E-03
Rps3a	ribosomal protein S3A	-1,524	7,675E-03
Dhx58	DEXH (Asp-Glu-X-His) box polypeptide 58	-1,524	4,207E-03
Rps6ka2	ribosomal protein S6 kinase polypeptide 2	-1,529	7,675E-03
Rnf130	ring finger protein 130	-1,529	7,675E-03
Sox13	SRY-box containing gene 13	-1,529	9,097E-03
Pik3r2	phosphatidylinositol 3-kinase regulatory subunit polypeptide 2 (p85 beta)	-1,531	5,124E-03
Art4	ADP-ribosyltransferase 4	-1,531	5,293E-03
Smarcd1	SWI/SNF related matrix associated actin dependent regulator of chromatin subfamily d member 1	-1,536	6,715E-03
Zfp219	zinc finger protein 219	-1,536	8,185E-03
4930473A06Rik	RIKEN cDNA 4930473A06 gene	-1,536	6,810E-03
Acadsb	acyl-Coenzyme A dehydrogenase short/branched chain	-1,536	6,673E-03
Scamp1	secretory carrier membrane protein 1	-1,536	3,905E-03
Dap3	death associated protein 3	-1,538	6,754E-03
Pex5	peroxisomal biogenesis factor 5	-1,538	5,518E-03
Syt11	synaptotagmin XI	-1,538	8,577E-03
Atpif1	ATPase inhibitory factor 1	-1,543	3,742E-03
Ivd	isovaleryl coenzyme A dehydrogenase	-1,543	8,566E-03
Naip5	NLR family apoptosis inhibitory protein 5	-1,546	7,577E-03
Man1a	mannosidase 1 alpha	-1,546	8,190E-03
BC005685	cDNA sequence BC005685	-1,546	4,249E-03
Cttn	cortactin	-1,548	7,412E-03
Abcg1	ATP-binding cassette sub-family G (WHITE) member 1	-1,548	8,234E-03
Btg1	B-cell translocation gene 1 anti-proliferative	-1,550	4,144E-03
Phlpp1	PH domain and leucine rich repeat protein phosphatase 1	-1,550	3,082E-03
Rasgrf2	RAS protein-specific guanine nucleotide-releasing factor 2	-1,553	4,553E-03
Btk	Bruton agammaglobulinemia tyrosine kinase	-1,553	4,368E-03
Rtkn	rhotekin	-1,555	7,530E-03
Tm7sf3	transmembrane 7 superfamily member 3	-1,555	3,279E-03
Zfp354c	zinc finger protein 354C	-1,555	5,906E-03
Cyth2	cytohesin 2	-1,558	4,876E-03
3110007F17Rik	RIKEN cDNA 3110007F17 gene	-1,558	6,349E-03
Gm525	predicted gene 525	-1,558	5,545E-03
Arid5b	AT rich interactive domain 5B (MRF1-like)	-1,560	5,116E-03
Stxbp4	syntaxin binding protein 4	-1,565	5,863E-03
Pex6	peroxisomal biogenesis factor 6	-1,565	5,416E-03
Ldoc1l	leucine zipper down-regulated in cancer 1-like	-1,565	6,956E-03
Srl	sarcalumenin	-1,565	3,386E-03
Pag1	phosphoprotein associated with glycosphingolipid microdomains 1	-1,565	7,075E-03
Ulk1	Unc-51 like kinase 1 (C. elegans)	-1,567	5,293E-03
St6galnac5	ST6 (alpha-N-acetyl-neuraminyl-23-beta-galactosyl-13)-N-acetylgalactosaminide alpha-26-sialyltransferase 5	-1,567	4,434E-03
Hdgfrp3	hepatoma-derived growth factor related protein 3	-1,567	6,880E-03
Zfp229	zinc finger protein	-1,567	5,898E-03
Dip2b	DIP2 disco-interacting protein 2 homolog B (Drosophila)	-1,567	3,565E-03

Mir181a-2		-1,572	9,090E-03
Pcca	propionyl-Coenzyme A carboxylase alpha polypeptide	-1,572	5,192E-03
Pip4k2a	phosphatidylinositol-5-phosphate 4-kinase type II alpha	-1,577	6,204E-03
Ern1	endoplasmic reticulum (ER) to nucleus signalling 1	-1,580	6,287E-03
Plcg1	phospholipase C gamma 1	-1,580	2,939E-03
Zc4h2	zinc finger C4H2 domain containing	-1,580	4,478E-03
Cdc20b	cell division cycle 20 homolog B (S. cerevisiae)	-1,580	6,975E-03
Tox3	TOX high mobility group box family member 3	-1,580	5,545E-03
Slc25a35	solute carrier family 25 member 35	-1,582	7,273E-03
Ncf2	neutrophil cytosolic factor 2	-1,582	4,482E-03
Frat2	frequently rearranged in advanced T-cell lymphomas 2	-1,585	2,980E-03
Gm17006	T cell receptor alpha variable 13-4/DV7	-1,585	5,892E-03
Ston1	stonin 1	-1,585	4,478E-03
Cd69	CD69 antigen	-1,587	8,232E-03
Ak4	adenylate kinase 3-like 1	-1,587	4,242E-03
5730471H19Rik	RIKEN cDNA 5730471H19 gene	-1,590	3,509E-03
Sema4c	sema domain immunoglobulin domain (Ig) transmembrane domain (TM) and short cytoplasmic domain (semaphorin) 4C	-1,592	6,636E-03
Gm16515	gene trap locus F3b	-1,592	3,398E-03
Dnalc1	dynein axonemal light chain 1	-1,592	5,135E-03
Kdm6b	KDM1 lysine (K)-specific demethylase 6B	-1,595	7,675E-03
Sord	sorbitol dehydrogenase	-1,595	3,942E-03
Ncs1	frequenin homolog (Drosophila)	-1,597	3,400E-03
Gstt2	glutathione S-transferase theta 2	-1,597	9,091E-03
Ifnar1	interferon (alpha and beta) receptor 1	-1,600	2,080E-03
Atf7ip	activating transcription factor 7 interacting protein	-1,600	3,547E-03
Mospd1	motile sperm domain containing 1	-1,600	5,577E-03
Anks1	ankyrin repeat and SAM domain containing 1	-1,603	6,673E-03
Stc1	stanniocalcin 1	-1,603	7,885E-03
Ptpn12	protein tyrosine phosphatase non-receptor type 12	-1,603	3,753E-03
Zswim4	zinc finger SWIM domain containing 4	-1,605	1,978E-03
Csrnp2	cysteine-serine-rich nuclear protein 2	-1,605	4,172E-03
Rundc3b	RUN domain containing 3B	-1,605	6,126E-03
Auh	AU RNA binding protein/enoyl-coenzyme A hydratase	-1,605	5,416E-03
Chd3	chromodomain helicase DNA binding protein 3	-1,605	6,754E-03
Kctd1	potassium channel tetramerisation domain containing 1	-1,605	2,394E-03
Plscr3	phospholipid scramblase 3	-1,608	3,686E-03
Stap1	signal transducing adaptor family member 1	-1,610	5,116E-03
Stxbp4	syntaxin binding protein 4	-1,610	6,810E-03
Frmd4b	FERM domain containing 4B	-1,610	2,578E-03
Fam125b	family with sequence similarity 125 member B	-1,610	4,947E-03
Rtkn2	rhotekin 2	-1,610	7,675E-03
Abtb1	ankyrin repeat and BTB (POZ) domain containing 1	-1,610	2,873E-03
Aff1	AF4/FMR2 family member 1	-1,610	5,622E-03
Wdr19	WD repeat domain 19	-1,610	6,502E-03
Ip6k2	inositol hexaphosphate kinase 2	-1,613	2,988E-03
Sord	sorbitol dehydrogenase	-1,613	4,020E-03
F13a1	coagulation factor XIII A1 subunit	-1,613	7,084E-03
Arrdc4	arrestin domain containing 4	-1,613	2,394E-03
Cyth3	cytohesin 3	-1,616	3,629E-03

Emilin1	elastin microfibril interfacer 1	**-1,618**	9,040E-03
Ilvbl	ilvB (bacterial acetolactate synthase)-like	**-1,621**	3,924E-03
Rnf125	ring finger protein 125	**-1,621**	6,754E-03
Foxn3	forkhead box N3	**-1,621**	1,671E-03
Eef2k	eukaryotic elongation factor-2 kinase	**-1,623**	6,975E-03
Angptl2	angiopoietin-like 2	**-1,623**	3,924E-03
Ralgps2	Ral GEF with PH domain and SH3 binding motif 2	**-1,623**	2,826E-03
Sh3bp2	SH3-domain binding protein 2	**-1,623**	1,882E-03
Gtpbp2	GTP binding protein 2	**-1,626**	2,742E-03
Idh1	isocitrate dehydrogenase 1 (NADP+) soluble	**-1,626**	6,975E-03
Lpin1	lipin 1	**-1,629**	1,511E-03
Zfp608	zinc finger protein 608	**-1,631**	3,606E-03
Dusp2	dual specificity phosphatase 2	**-1,631**	1,782E-03
P2rx7	purinergic receptor P2X ligand-gated ion channel 7	**-1,634**	1,689E-03
Map4k4	mitogen-activated protein kinase kinase kinase kinase 4	**-1,634**	2,612E-03
As3mt	arsenic (+3 oxidation state) methyltransferase	**-1,634**	7,108E-03
Pitpnc1	phosphatidylinositol transfer protein cytoplasmic 1	**-1,637**	3,453E-03
Glb1l	galactosidase beta 1-like	**-1,637**	4,041E-03
Arvcf	armadillo repeat gene deleted in velo-cardio-facial syndrome	**-1,637**	1,424E-03
Snn	stannin	**-1,639**	3,629E-03
Aff4	AF4/FMR2 family member 4	**-1,639**	2,531E-03
Dnahc8	dynein axonemal heavy chain 8	**-1,642**	6,120E-03
LOC641050	hypothetical protein LOC641050	**-1,642**	3,120E-03
Btg2	B-cell translocation gene 2 anti-proliferative	**-1,645**	1,736E-03
Galc	galactosylceramidase	**-1,645**	7,567E-03
Atp13a2	ATPase type 13A2	**-1,645**	6,949E-03
Ccdc122	coiled-coil domain containing 122	**-1,645**	1,407E-03
Klhdc2	kelch domain containing 2	**-1,647**	8,239E-03
Cd93	CD93 antigen	**-1,647**	1,677E-03
Ston2	stonin 2	**-1,647**	3,095E-03
Akap6	A kinase (PRKA) anchor protein 6	**-1,650**	1,583E-03
Acss2	acyl-CoA synthetase short-chain family member 2	**-1,653**	3,942E-03
Peli1	pellino 1	**-1,656**	4,468E-03
Aim2	absent in melanoma 2	**-1,658**	3,660E-03
Shisa5	shisa homolog 5 (Xenopus laevis)	**-1,661**	2,461E-03
Cxxc5	CXXC finger 5	**-1,667**	4,158E-03
Maml2	mastermind like 2 (Drosophila)	**-1,667**	3,266E-03
Il21r	interleukin 21 receptor	**-1,669**	1,250E-03
Dnajb4	DnaJ (Hsp40) homolog subfamily B member 4	**-1,669**	5,517E-03
Pgap1	post-GPI attachment to proteins 1	**-1,669**	1,763E-03
Hist1h3d	histone cluster 1 H3d	**-1,669**	1,407E-03
Acvr1b	activin A receptor type 1B	**-1,669**	1,910E-03
Tpcn1	two pore channel 1	**-1,672**	4,472E-03
Ifih1	interferon induced with helicase C domain 1	**-1,675**	1,887E-03
Tnfrsf26	tumor necrosis factor receptor superfamily member 26	**-1,675**	1,221E-03
D930015E06Rik	RIKEN cDNA D930015E06 gene	**-1,675**	1,008E-03
Cbx2	chromobox homolog 2 (Drosophila Pc class)	**-1,678**	2,930E-03
Eif2ak3	eukaryotic translation initiation factor 2 alpha kinase 3	**-1,681**	1,081E-03
Patz1	POZ (BTB) and AT hook containing zinc finger 1	**-1,681**	1,418E-03

Gm13949	predicted gene 13949	-1,681	4,926E-03
Ptk7	PTK7 protein tyrosine kinase 7	-1,686	1,505E-03
Tcp11l2	t-complex 11 (mouse) like 2	-1,686	5,171E-03
Il9r	interleukin 9 receptor	-1,686	1,506E-03
Slc9a9	solute carrier family 9 (sodium/hydrogen exchanger) member 9	-1,686	4,947E-03
Dst	dystonin	-1,689	2,429E-03
Cmtm7	CKLF-like MARVEL transmembrane domain containing 7	-1,692	1,145E-03
Morn2	MORN repeat containing 2	-1,692	2,504E-03
Arhgef2	rho/rac guanine nucleotide exchange factor (GEF) 2	-1,692	1,532E-03
Map2k6	mitogen-activated protein kinase kinase 6	-1,695	1,133E-03
Bcl2l14	BCL2-like 14 (apoptosis facilitator)	-1,698	5,906E-03
Kcnrg	potassium channel regulator	-1,701	5,545E-03
Oxct1	3-oxoacid CoA transferase 1	-1,701	1,231E-03
Dyrk1b	dual-specificity tyrosine-(Y)-phosphorylation regulated kinase 1b	-1,706	3,136E-03
Gimap6	GTPase IMAP family member 6	-1,706	1,850E-03
Bcat1	branched chain aminotransferase 1 cytosolic	-1,709	1,250E-03
Frat1	frequently rearranged in advanced T-cell lymphomas	-1,712	1,079E-03
Ecm1	extracellular matrix protein 1	-1,712	3,425E-03
Lpar4	lysophosphatidic acid receptor 4	-1,715	1,515E-03
Pmepa1	prostate transmembrane protein androgen induced 1	-1,718	1,841E-03
Trav14-3	T-cell receptor alpha chain	-1,718	5,912E-03
Tm7sf2	transmembrane 7 superfamily member 2	-1,718	1,424E-03
Mapkapk2	MAP kinase-activated protein kinase 2	-1,721	1,528E-03
LOC625360	similar to 2-cell-stage variable group member 3	-1,724	5,311E-03
Gm4983	predicted gene 9282	-1,724	1,952E-03
Spint2	serine protease inhibitor Kunitz type 2	-1,724	1,377E-03
Mrgpre	MAS-related GPR member E	-1,724	1,250E-03
Sytl3	synaptotagmin-like 3	-1,727	7,190E-03
Ptpn22	protein tyrosine phosphatase non-receptor type 22 (lymphoid)	-1,730	1,448E-03
Hist1h2be	histone cluster 1 H1e	-1,730	1,710E-03
Als2	amyotrophic lateral sclerosis 2 (juvenile) homolog (human)	-1,733	6,862E-04
St8sia4	ST8 alpha-N-acetyl-neuraminide alpha-28-sialyltransferase 4	-1,736	5,154E-03
St6gal1	beta galactoside alpha 26 sialyltransferase 1	-1,736	1,448E-03
Wls	G protein-coupled receptor 177	-1,739	3,565E-03
Ppm1h	protein phosphatase 1H (PP2C domain containing)	-1,739	3,365E-03
Gata3	GATA binding protein 3	-1,739	6,786E-04
Cd200	CD200 antigen	-1,739	6,840E-04
Ptprf	protein tyrosine phosphatase receptor type F	-1,739	5,970E-03
Lpp	LIM domain containing preferred translocation partner in lipoma	-1,739	9,598E-04
Ifi27l1	interferon alpha-inducible protein 27 like 1	-1,742	1,138E-03
Gm13979	predicted gene OTTMUSG00000015223	-1,742	1,515E-03
Dnmt3a	DNA methyltransferase 3A	-1,748	7,101E-04
Baz2b	bromodomain adjacent to zinc finger domain 2B	-1,748	2,080E-03
Tfdp2	transcription factor Dp 2	-1,751	8,004E-04
Ppp2r3a	protein phosphatase 2 regulatory subunit B'' alpha	-1,754	3,317E-03
Spats2	spermatogenesis associated serine-rich 2	-1,754	1,645E-03
Tmem151b	transmembrane protein 151B	-1,761	5,016E-03
Gpx4	glutathione peroxidase 4	-1,761	6,956E-03
Bend5	BEN domain containing 5	-1,764	3,328E-03

Homer2	RIKEN cDNA 9330120H11 gene	-1,764	3,359E-03
Trim12a	tripartite motif-containing 12	-1,764	7,722E-03
Ddah2	dimethylarginine dimethylaminohydrolase 2	-1,767	1,145E-03
Fmnl3	formin-like 3	-1,767	7,899E-04
Adcy6	adenylate cyclase 6	-1,773	5,600E-03
Mtf2	metal response element binding transcription factor 2	-1,776	3,329E-03
Gimap4	GTPase IMAP family member 4	-1,776	7,957E-04
Psd3	pleckstrin and Sec7 domain containing 3	-1,779	3,328E-03
Bphl	biphenyl hydrolase-like (serine hydrolase breast epithelial mucin-associated antigen)	-1,779	6,092E-04
Tnfsf10	tumor necrosis factor (ligand) superfamily member 10	-1,783	2,608E-03
Gatsl2	GATS protein-like 2	-1,783	1,645E-03
Tmem67	transmembrane protein 67	-1,786	5,212E-03
Hmga2	high mobility group AT-hook 2	-1,786	1,588E-03
Trp53inp2	transformation related protein 53 inducible nuclear protein 2	-1,789	1,007E-03
Mir15b		-1,792	8,656E-03
Peci	peroxisomal delta3 delta2-enoyl-Coenzyme A isomerase	-1,792	2,939E-03
Litaf	LPS-induced TN factor	-1,795	5,004E-04
Ctxn1	cortexin 1	-1,802	2,418E-03
Lrrc32	leucine rich repeat containing 32	-1,802	5,125E-04
Gm13927	T-cell receptor alpha chain	-1,802	1,812E-03
Sfrp2	secreted frizzled-related protein 2	-1,802	2,800E-03
H2afv	H2A histone family member V	-1,805	3,328E-03
Pgap1	post-GPI attachment to proteins 1	-1,805	5,618E-03
Itm2c	integral membrane protein 2C	-1,808	3,082E-03
Ube2h	ubiquitin-conjugating enzyme E2H	-1,808	6,745E-04
Hsd17b11	hydroxysteroid (17-beta) dehydrogenase 11	-1,812	6,821E-04
Alpk1	alpha-kinase 1	-1,812	3,260E-03
Slamf1	signaling lymphocytic activation molecule family member 1	-1,815	2,827E-03
Padi2	peptidyl arginine deiminase type II	-1,818	7,713E-04
Tubb2b	tubulin beta 2B	-1,818	2,988E-03
Nrgn	neurogranin	-1,821	2,326E-03
C630004H02Rik	RIKEN cDNA C630004H02 gene	-1,821	4,145E-04
Mrgpra9	predicted gene EG668725	-1,821	1,159E-03
Trf	transferrin	-1,825	9,267E-04
Tmc5	transmembrane channel-like gene family 5	-1,825	1,019E-03
Egr1	early growth response 1	-1,828	1,397E-03
Enah	enabled homolog (Drosophila)	-1,832	1,536E-03
Crip2	cysteine rich protein 2	-1,835	3,660E-03
Dclk2	doublecortin-like kinase 2	-1,835	6,142E-03
Ccng2	cyclin G2	-1,838	1,133E-03
Tgfb3	transforming growth factor beta 3	-1,838	7,324E-04
Ick	intestinal cell kinase	-1,838	2,993E-04
Slc7a8	solute carrier family 7 (cationic amino acid transporter y+ system) member 8	-1,842	6,948E-04
Usp11	ubiquitin specific peptidase 11	-1,845	6,829E-04
Acy3	aspartoacylase (aminoacylase) 3	-1,848	6,461E-04
Hspb11	heat shock protein family B (small) member 11	-1,848	9,120E-03
Zscan2	zinc finger and SCAN domain containing 2	-1,848	5,414E-04
Parp12	poly (ADP-ribose) polymerase family member 12	-1,848	5,135E-03

Rapgef3	Rap guanine nucleotide exchange factor (GEF) 3	-1,848	1,053E-03
A530021J07Rik	Riken cDNA A530021J07 gene	-1,852	4,240E-04
Zfp821	zinc finger protein 821	-1,852	3,165E-04
Ube2h	ubiquitin-conjugating enzyme E2H	-1,852	5,266E-04
Hivep3	human immunodeficiency virus type I enhancer binding protein 3	-1,852	4,441E-03
Cd96	CD96 antigen	-1,855	1,453E-03
Myb	myeloblastosis oncogene	-1,855	7,729E-04
Rapgef2	Rap guanine nucleotide exchange factor (GEF) 2	-1,855	1,391E-03
Adssl1	adenylosuccinate synthetase like 1	-1,855	5,316E-03
Sema7a	sema domain immunoglobulin domain (Ig) and GPI membrane anchor (semaphorin) 7A	-1,855	1,511E-03
Prkcq	protein kinase C theta	-1,859	3,517E-04
Pyhin1	pyrin and HIN domain family member 1	-1,859	7,121E-04
Mir181a-1		-1,866	3,605E-04
Bace1	beta-site APP cleaving enzyme 1	-1,866	3,081E-04
Slfn5	schlafen 5	-1,866	6,816E-04
Dgke	diacylglycerol kinase epsilon	-1,869	4,158E-03
Papss2	3'-phosphoadenosine 5'-phosphosulfate synthase 2	-1,869	1,145E-03
Pak1	p21 protein (Cdc42/Rac)-activated kinase 1	-1,873	6,816E-04
Ifit3	interferon-induced protein with tetratricopeptide repeats 3	-1,873	8,182E-04
Chchd3	coiled-coil-helix-coiled-coil-helix domain containing 3	-1,873	7,910E-04
Tcf4	transcription factor 4	-1,880	1,110E-03
Kdelc1	KDEL (Lys-Asp-Glu-Leu) containing 1	-1,883	9,682E-04
Lrmp	lymphoid-restricted membrane protein	-1,887	3,082E-03
Gpx4	glutathione peroxidase 4	-1,887	3,259E-03
D18Ertd653e	DNA segment Chr 18 ERATO Doi 653 expressed	-1,890	3,606E-04
Snrpe	small nuclear ribonucleoprotein E	-1,890	1,931E-03
Slc2a3	solute carrier family 2 (facilitated glucose transporter) member 3	-1,894	2,636E-04
H2-DMa	histocompatibility 2 class II locus DMa	-1,901	5,696E-04
Terc	telomerase RNA component	-1,901	4,321E-03
B3gnt2	UDP-GlcNAc:betaGal beta-13-N-acetylglucosaminyltransferase 2	-1,905	6,043E-03
Galnt6	UDP-N-acetyl-alpha-D-galactosamine:polypeptide N-acetylgalactosaminyltransferase 6	-1,905	5,029E-04
Spata6	spermatogenesis associated 6	-1,908	4,496E-04
Nr4a3	nuclear receptor subfamily 4 group A member 3	-1,908	1,085E-03
Plscr1	phospholipid scramblase 1	-1,908	1,231E-03
Arhgap42	RIKEN cDNA 9030420J04 gene	-1,908	2,958E-04
Gm10688	predicted gene 10688	-1,912	7,060E-03
Hpcal1	hippocalcin-like 1	-1,912	7,720E-04
St6gal1	beta galactoside alpha 26 sialyltransferase 1	-1,916	5,016E-03
Cd59a	CD59a antigen	-1,916	4,368E-03
Hist1h2bc	histone cluster 1 H2bc	-1,919	2,271E-03
Acss1	acyl-CoA synthetase short-chain family member 1	-1,923	3,667E-04
Ptgfrn	prostaglandin F2 receptor negative regulator	-1,923	1,358E-03
Tubb2a	tubulin beta 2A	-1,927	3,492E-03
Casp6	caspase 6	-1,927	5,906E-04
Slc43a1	solute carrier family 43 member 1	-1,931	8,297E-04
Amz2	archaelysin family metallopeptidase 2	-1,938	2,570E-04

Sh3pxd2a	RIKEN cDNA 2310014D11 gene	-1,942	4,064E-04
Ptprk	protein tyrosine phosphatase receptor type K	-1,942	9,844E-04
Homer2	homer homolog 2 (Drosophila)	-1,942	2,013E-03
Prkca	protein kinase C alpha	-1,942	6,862E-04
Aim1	absent in melanoma 1	-1,946	1,186E-03
Galnt10	UDP-N-acetyl-alpha-D-galactosamine:polypeptide N-acetylgalactosaminyltransferase 10	-1,946	1,710E-03
Fbxl20	F-box and leucine-rich repeat protein 20	-1,949	6,465E-04
Dusp6	dual specificity phosphatase 6	-1,949	4,127E-04
Prdx2	peroxiredoxin 2	-1,949	1,200E-03
Dpp4	dipeptidylpeptidase 4	-1,957	4,518E-04
Tspan31	tetraspanin 31	-1,957	5,340E-04
Igf2	insulin-like growth factor 2	-1,961	6,176E-04
Ccl3	chemokine (C-C motif) ligand 3	-1,965	7,272E-04
Vangl2	vang-like 2 (van gogh Drosophila)	-1,965	2,577E-04
Sykb	spleen tyrosine kinase	-1,969	6,574E-04
Gm16489	predicted gene 16489	-1,972	6,169E-03
2010107G12Rik	RIKEN cDNA 2010107G12 gene	-1,976	2,691E-04
5730559C18Rik	RIKEN cDNA 5730559C18 gene	-1,984	4,756E-03
Tspan13	tetraspanin 13	-1,984	1,371E-04
Csnk1e	casein kinase 1 epsilon	-1,984	2,577E-04
Rxfp1	relaxin/insulin-like family peptide receptor 1	-1,984	6,502E-03
Gm13926	predicted gene 13926	-1,984	2,715E-04
Galnt3	UDP-N-acetyl-alpha-D-galactosamine:polypeptide N-acetylgalactosaminyltransferase 3	-1,988	1,689E-04
Cbr1	carbonyl reductase 1	-1,988	5,125E-04
Ppm1l	protein phosphatase 1 (formerly 2C)-like	-1,988	3,484E-04
AI452195	expressed sequence AI452195	-1,992	4,120E-04
Iqch	IQ motif containing H	-1,996	2,820E-03
Cd1d1	CD1d1 antigen	-1,996	2,977E-03
Ldhb	lactate dehydrogenase B	-2,000	2,451E-03
Trav14d-1		-2,004	2,667E-04
Ets2	E26 avian leukemia oncogene 2 3' domain	-2,008	1,053E-03
Gk5	glycerol kinase 5 (putative)	-2,008	7,172E-04
Ypel2	yippee-like 2 (Drosophila)	-2,012	1,266E-04
Mr1	major histocompatibility complex class I-related	-2,016	1,371E-04
Wsb2	WD repeat and SOCS box-containing 2	-2,016	1,382E-04
Syt13	synaptotagmin XIII	-2,020	6,491E-04
Ephx1	epoxide hydrolase 1 microsomal	-2,020	6,745E-04
Prkcb	protein kinase C beta	-2,024	1,069E-04
Nfkbie	nuclear factor of kappa light polypeptide gene enhancer in B-cells inhibitor epsilon	-2,053	3,758E-04
Rgs10	regulator of G-protein signalling 10	-2,058	3,814E-04
Snord61	small nucleolar RNA C/D box 61	-2,058	3,651E-04
Bach1	BTB and CNC homology 1	-2,058	1,358E-04
Sestd1	SEC14 and spectrin domains 1	-2,062	7,885E-03
Slc45a3	solute carrier family 45 member 3	-2,066	1,523E-04
Egr2	early growth response 2	-2,066	1,006E-03
Ift80	intraflagellar transport 80 homolog (Chlamydomonas)	-2,066	2,221E-04
Gm6907	predicted gene 6907	-2,070	2,159E-04

Dgkg	diacylglycerol kinase gamma	-2,070	3,750E-04
Bpgm	23-bisphosphoglycerate mutase	-2,070	2,751E-03
Uaca	uveal autoantigen with coiled-coil domains and ankyrin repeats	-2,075	1,515E-03
Prdx2	peroxiredoxin 2	-2,079	8,954E-05
Gem	GTP binding protein (gene overexpressed in skeletal muscle)	-2,079	1,475E-04
Prcp	prolylcarboxypeptidase (angiotensinase C)	-2,079	1,655E-04
D430041D05Rik	RIKEN cDNA D430041D05 gene	-2,083	5,340E-04
Cd2	CD2 antigen	-2,083	1,382E-04
Hist2h2be	histone cluster 2 H2be	-2,083	2,841E-04
Cdyl2	chromodomain protein Y chromosome-like 2	-2,088	4,825E-04
Dmwd	dystrophia myotonica-containing WD repeat motif	-2,092	1,529E-04
Rbpms	RNA binding protein gene with multiple splicing	-2,096	1,646E-04
Adamts6	a disintegrin-like and metallopeptidase (reprolysin type) with thrombospondin type 1 motif 6	-2,096	6,210E-03
Prkar2b	protein kinase cAMP dependent regulatory type II beta	-2,096	3,306E-04
Ldhb	lactate dehydrogenase B	-2,096	3,795E-04
Dzip1	DAZ interacting protein 1	-2,096	2,153E-04
Adora2a	adenosine A2a receptor	-2,096	1,118E-04
Nkg7	natural killer cell group 7 sequence	-2,101	7,899E-04
Slc35b4	solute carrier family 35 member B4	-2,110	1,448E-03
Dapk1	death associated protein kinase 1	-2,110	3,386E-04
Bivm	basic immunoglobulin-like variable motif containing	-2,114	1,929E-04
Dnajc6	DnaJ (Hsp40) homolog subfamily C member 6	-2,119	1,824E-04
Ms4a6c	membrane-spanning 4-domains subfamily A member 6C	-2,119	3,021E-04
Zfp36l2	zinc finger protein 36 C3H type-like 2	-2,128	4,116E-04
Calcoco1	calcium binding and coiled coil domain 1	-2,141	3,081E-04
Rgs12	regulator of G-protein signaling 12	-2,146	6,564E-05
Nsg2	neuron specific gene family member 2	-2,146	3,105E-03
Pdlim4	PDZ and LIM domain 4	-2,151	1,596E-04
Cdh17	cadherin 17	-2,151	5,212E-03
Ano6	anoctamin 6	-2,160	7,062E-05
Idh2	isocitrate dehydrogenase 2 (NADP+) mitochondrial	-2,169	1,186E-04
Rnf157	ring finger protein 157	-2,174	8,128E-04
Snord118	small nucleolar RNA C/D box 118	-2,174	2,931E-03
Spib	Spi-B transcription factor (Spi-1/PU.1 related)	-2,183	2,653E-03
Aff3	AF4/FMR2 family member 3	-2,193	5,857E-04
Lck	lymphocyte protein tyrosine kinase	-2,203	1,452E-04
Plscr1	phospholipid scramblase 1	-2,212	3,256E-04
Parp8	poly (ADP-ribose) polymerase family member 8	-2,217	3,256E-04
Rsad2	radical S-adenosyl methionine domain containing 2	-2,217	1,689E-04
Lrp12	low density lipoprotein-related protein 12	-2,227	5,583E-05
Ifit1	interferon-induced protein with tetratricopeptide repeats 1	-2,227	1,129E-03
Nck2	non-catalytic region of tyrosine kinase adaptor protein 2	-2,232	5,475E-05
Wsb2	WD repeat and SOCS box-containing 2	-2,237	6,780E-05
D430041D05Rik	RIKEN cDNA D430041D05 gene	-2,237	5,468E-05
Stom	stomatin	-2,247	4,206E-04
Card6	caspase recruitment domain family member 6	-2,257	1,320E-04
Casp3	caspase 3	-2,262	2,143E-04
Mex3b	mex3 homolog B (C. elegans)	-2,268	8,718E-05

Mctp2	multiple C2 domains transmembrane 2	-2,273	6,341E-03
Rgs2	regulator of G-protein signaling 2	-2,273	8,453E-05
Gfi1	growth factor independent 1	-2,278	2,252E-04
Spns3	spinster homolog 3 (Drosophila)	-2,283	4,836E-05
Tcrb-J	T-cell receptor beta joining region	-2,283	6,461E-04
Rnu1b1	U1b1 small nuclear RNA	-2,288	7,113E-03
Tcf7	transcription factor 7 T-cell specific	-2,294	3,491E-04
Gm10483	predicted gene 10483	-2,299	5,208E-04
Gbp9	cDNA sequence BC057170	-2,299	1,169E-04
Pik3ip1	phosphoinositide-3-kinase interacting protein 1	-2,299	4,063E-05
4930427A07Rik	RIKEN cDNA 4930427A07 gene	-2,299	1,131E-04
Cxcr4	chemokine (C-X-C motif) receptor 4	-2,304	6,270E-05
Tspan6	tetraspanin 6	-2,304	5,735E-05
Nck2	non-catalytic region of tyrosine kinase adaptor protein 2	-2,304	1,677E-04
Uqcrq	ubiquinol-cytochrome c reductase complex III subunit VII	-2,309	8,853E-04
BC048355	cDNA sequence BC048355	-2,309	1,184E-03
Mex3a	mex3 homolog A (C. elegans)	-2,315	1,371E-04
Il6st	interleukin 6 signal transducer	-2,315	4,589E-05
Satb1	special AT-rich sequence binding protein 1	-2,315	1,336E-04
Wfs1	Wolfram syndrome 1 homolog (human)	-2,320	6,461E-04
Fam169b	family with sequence similarity 169 member B	-2,326	2,334E-04
Tet1	tet oncogene 1	-2,326	3,016E-05
Tlr12	toll-like receptor 12	-2,326	2,577E-04
Tet1	tet oncogene 1	-2,331	3,001E-05
Ptger4	prostaglandin E receptor 4 (subtype EP4)	-2,336	2,199E-04
A130050O07Rik	RIKEN cDNA A130050O07 gene	-2,347	2,823E-03
Gpr97	G protein-coupled receptor 97	-2,353	1,459E-04
Rhoh	ras homolog gene family member H	-2,392	3,857E-05
Arhgap32	Rho GTPase-activating protein	-2,404	1,123E-04
Akr1c13	aldo-keto reductase family 1 member C13	-2,404	1,023E-04
St3gal2	ST3 beta-galactoside alpha-23-sialyltransferase 2	-2,410	3,025E-05
Endou	placental protein 11 related	-2,415	5,144E-05
Ublcp1	ubiquitin-like domain containing CTD phosphatase 1	-2,415	2,165E-05
Kcnn4	potassium intermediate/small conductance calcium-activated channel subfamily N member 4	-2,421	3,439E-05
Trib2	tribbles homolog 2 (Drosophila)	-2,421	2,042E-05
Rhobtb3	Rho-related BTB domain containing 3	-2,427	1,336E-04
Rab33a	RAB33A member of RAS oncogene family	-2,433	3,476E-03
Gpx8	glutathione peroxidase 8 (putative)	-2,433	7,564E-04
Tns3	tensin 3	-2,445	4,306E-05
Ptgir	prostaglandin I receptor (IP)	-2,457	2,106E-04
Klhl24	kelch-like 24 (Drosophila)	-2,463	7,708E-05
Lass6	LAG1 homolog ceramide synthase 6	-2,463	2,703E-05
Sort1	sortilin 1	-2,475	5,581E-05
Pgpep1l	RIKEN cDNA C330024D12 gene	-2,481	1,423E-04
Lztfl1	leucine zipper transcription factor-like 1	-2,488	3,983E-05
Mir181b-2		-2,494	3,025E-05
1110028C15Rik	RIKEN cDNA 1110028C15 gene	-2,500	7,949E-05
Plxdc2	plexin domain containing 2	-2,506	1,651E-05

Capn5	calpain 5	-2,513	2,577E-04
Tet1	tet oncogene 1	-2,513	1,650E-04
Ddx60	DEAD (Asp-Glu-Ala-Asp) box polypeptide 60	-2,519	2,207E-04
Gpr125	G protein-coupled receptor 125	-2,532	2,182E-05
Gm885	predicted gene 885	-2,538	1,191E-04
Mgst2	microsomal glutathione S-transferase 2	-2,538	6,780E-05
Rab27b	RAB27b member RAS oncogene family	-2,545	4,072E-04
Lig4	ligase IV DNA ATP-dependent	-2,564	7,019E-05
Lztfl1	leucine zipper transcription factor-like 1	-2,571	9,314E-04
Ccdc109b	coiled-coil domain containing 109B	-2,577	2,823E-03
Slc14a1	solute carrier family 14 (urea transporter) member 1	-2,577	2,368E-05
Ccr4	chemokine (C-C motif) receptor 4	-2,584	3,905E-05
Ifitm3	interferon induced transmembrane protein 3	-2,591	1,145E-03
Bach2	BTB and CNC homology 2	-2,597	3,382E-04
Aldh2	aldehyde dehydrogenase 2 mitochondrial	-2,604	3,570E-05
Colq	collagen-like tail subunit (single strand of homotrimer) of asymmetric acetylcholinesterase	-2,618	3,606E-04
Cnn3	calponin 3 acidic	-2,618	6,493E-05
BC106179	cDNA sequence BC106179	-2,639	7,899E-04
Hmga2	RIKEN cDNA 1700006J14 gene	-2,639	1,120E-04
Tnfrsf9	tumor necrosis factor receptor superfamily member 9	-2,646	4,078E-05
Gyg	glycogenin	-2,660	8,960E-05
Il17rb	interleukin 17 receptor B	-2,667	2,710E-04
Gm13895	predicted gene 13895	-2,674	7,708E-05
Lef1	lymphoid enhancer binding factor 1	-2,695	1,174E-05
Dzip1	DAZ interacting protein 1	-2,703	2,182E-05
Trp53inp1	transformation related protein 53 inducible nuclear protein 1	-2,717	7,572E-05
Gsn	gelsolin	-2,732	2,369E-04
Fyb	FYN binding protein	-2,732	5,059E-05
Art2b	ADP-ribosyltransferase 2b	-2,740	5,067E-04
Rag2	recombination activating gene 2	-2,740	8,070E-03
Prssl1	protease serine-like 1	-2,747	2,640E-04
Gm6033	predicted gene EG547328	-2,755	1,724E-05
Tsc22d1	TSC22 domain family member 1	-2,762	2,422E-05
Klf3	Kruppel-like factor 3 (basic)	-2,762	8,672E-06
Rap1gap2	GTPase activating RANGAP domain-like 4	-2,778	1,968E-05
Ptpn14	protein tyrosine phosphatase non-receptor type 14	-2,778	2,841E-04
St8sia1	ST8 alpha-N-acetyl-neuraminide alpha-28-sialyltransferase 1	-2,778	1,710E-05
Ms4a4b	membrane-spanning 4-domains subfamily A member 4B	-2,786	1,464E-03
Rasgrp4	RAS guanyl releasing protein 4	-2,793	4,063E-05
Esm1	endothelial cell-specific molecule 1	-2,793	2,435E-04
Kdm5b	lysine (K)-specific demethylase 5B	-2,793	1,517E-05
Ubash3a	ubiquitin associated and SH3 domain containing A	-2,809	1,120E-04
Smo	smoothened homolog (Drosophila)	-2,817	3,182E-05
Tubb2b	tubulin beta 2B	-2,833	2,254E-03
Pld4	phospholipase D family member 4	-2,874	6,300E-05
Tmem120b	transmembrane protein 120B	-2,899	7,160E-06
Spo11	sporulation protein meiosis-specific SPO11 homolog (S. cerevisiae)	-2,899	1,400E-04
Ssbp2	single-stranded DNA binding protein 2	-2,915	3,016E-05
Khdc1a	KH domain containing 1A	-2,959	8,182E-04

Id3	inhibitor of DNA binding 3	-2,959	2,610E-05
Itgb3	integrin beta 3	-2,967	3,016E-05
H2afy2	H2A histone family member Y2	-2,976	5,769E-06
Hfm1	HFM1 ATP-dependent DNA helicase homolog (S. cerevisiae)	-2,994	1,386E-04
Inpp4b	inositol polyphosphate-4-phosphatase type II	-3,003	1,382E-04
Tspan9	tetraspanin 9	-3,012	1,139E-05
Clec12a	C-type lectin domain family 12 member a	-3,058	1,131E-04
Cx3cr1	chemokine (C-X3-C) receptor 1	-3,067	3,025E-05
Dusp10	dual specificity phosphatase 10	-3,086	3,025E-05
Gm10384	predicted gene 10384	-3,096	1,707E-05
Marcksl1	MARCKS-like 1	-3,106	3,710E-06
Cpt1a	carnitine palmitoyltransferase 1a liver	-3,135	3,870E-06
Mpp4	membrane protein palmitoylated 4 (MAGUK p55 subfamily member 4)	-3,145	9,039E-06
Itih5	inter-alpha (globulin) inhibitor H5	-3,155	1,724E-05
Cnn3	calponin 3 acidic	-3,155	6,129E-05
Hes1	hairy and enhancer of split 1 (Drosophila)	-3,185	8,950E-06
Pecam1	platelet/endothelial cell adhesion molecule 1	-3,205	3,795E-06
Dgka	diacylglycerol kinase alpha	-3,226	1,230E-05
Chn2	chimerin (chimaerin) 2	-3,247	4,934E-05
Kit	kit oncogene	-3,247	3,970E-06
Ifitm2	interferon induced transmembrane protein 2	-3,257	5,739E-04
Mir181b-1		-3,268	2,710E-04
Ccr7	chemokine (C-C motif) receptor 7	-3,279	2,336E-05
H19	H19 fetal liver mRNA	-3,333	8,470E-04
Btla	B and T lymphocyte associated	-3,344	3,857E-05
Robo1	roundabout homolog 1 (Drosophila)	-3,344	1,724E-05
Tmem121	transmembrane protein 121	-3,401	2,413E-06
Lgals9	lectin galactose binding soluble 9	-3,413	5,306E-06
Fam40b	family with sequence similarity 40 member B	-3,413	4,964E-06
Actn1	actinin alpha 1	-3,436	2,208E-05
Calcrl	calcitonin receptor-like	-3,448	4,716E-06
Slc16a5	solute carrier family 16 (monocarboxylic acid transporters) member 5	-3,534	1,131E-04
Il10ra	interleukin 10 receptor alpha	-3,546	8,441E-06
Gm13892	predicted gene 13892	-3,559	1,024E-05
Trim13	tripartite motif-containing 13	-3,584	3,744E-06
Cd27	CD27 antigen	-3,597	2,919E-06
5830411N06Rik	RIKEN cDNA 5830411N06 gene	-3,623	5,211E-06
Mcoln3	mucolipin 3	-3,623	1,654E-06
Gdpd1	glycerophosphodiester phosphodiesterase domain containing 1	-3,636	3,795E-06
Fads2	fatty acid desaturase 2	-3,704	3,047E-04
Nr4a1	nuclear receptor subfamily 4 group A member 1	-3,788	5,156E-05
Sh2d1a	SH2 domain protein 1A	-3,802	1,399E-05
Usp18	ubiquitin specific peptidase 18	-3,817	4,716E-06
Angpt1	angiopoietin 1	-3,846	2,773E-06
Gm13926	predicted gene 13926	-3,906	3,698E-06
Trim30d	expressed sequence AI451617	-4,000	1,045E-05
2810408A11Rik	RIKEN cDNA 2810408A11 gene	-4,000	1,947E-05

Gm13894	predicted gene 13894	-4,184	3,008E-06
Etv5	ets variant gene 5	-4,255	7,953E-07
Slc22a3	solute carrier family 22 (organic cation transporter) member 3	-4,255	1,099E-06
Etv5	ets variant gene 5	-4,310	8,629E-07
Prkch	protein kinase C eta	-4,386	2,182E-05
H19	H19 fetal liver mRNA	-4,464	3,009E-05
Aqp11	aquaporin 11	-4,484	3,805E-06
Akr1c12	aldo-keto reductase family 1 member C12	-4,525	1,015E-06
Ifitm2	interferon induced transmembrane protein 2	-4,566	3,281E-05
Gpr56	G protein-coupled receptor 56	-4,785	8,954E-05
Ms4a6b	membrane-spanning 4-domains subfamily A member 6B	-4,785	3,681E-06
Hectd2	HECT domain containing 2	-4,854	6,305E-06
Gpr174	G protein-coupled receptor 174	-4,902	1,174E-05
Sox4	SRY-box containing gene 4	-4,926	1,015E-06
Ccr8	chemokine (C-C motif) receptor 8	-4,926	2,660E-06
Ifitm2	interferon induced transmembrane protein 2	-5,051	2,512E-05
Itga4	integrin alpha 4	-5,102	1,222E-05
Mpzl2	myelin protein zero-like 2	-5,236	2,241E-03
I830077J02Rik	RIKEN cDNA I830077J02 gene	-5,236	1,880E-06
Scin	scinderin	-5,464	2,735E-05
Sstr2	somatostatin receptor 2	-5,495	6,535E-07
Fbp1	fructose bisphosphatase 1	-5,525	2,839E-05
Gpr83	G protein-coupled receptor 83	-5,525	1,458E-06
Folr4	folate receptor 4 (delta)	-5,682	7,953E-07
Bex6	brain expressed gene 6	-5,882	7,597E-07
Il2ra	interleukin 2 receptor alpha chain	-6,061	4,918E-03
Gria3	glutamate receptor ionotropic AMPA3 (alpha 3)	-6,250	7,597E-07
Gfra1	glial cell line derived neurotrophic factor family receptor alpha 1	-6,410	3,526E-07
Arpp21	cyclic AMP-regulated phosphoprotein 21	-6,452	2,660E-06
Il12rb2	interleukin 12 receptor beta 2	-6,452	3,008E-06
Gzma	granzyme A	-6,993	2,880E-06
Cd28	CD28 antigen	-7,299	3,008E-06
Trat1	T cell receptor associated transmembrane adaptor 1	-8,130	2,071E-06
Gm6683	predicted gene 6683	-8,264	3,832E-06
Sell	selectin lymphocyte	-8,333	3,526E-07
Igfbp4	insulin-like growth factor binding protein 4	-8,333	6,555E-07
Slc15a1	solute carrier family 15 (oligopeptide transporter) member 1	-8,403	4,966E-07
Gm16591		-8,547	4,966E-07
Marcks	myristoylated alanine rich protein kinase C substrate	-8,547	3,526E-07
Themis	thymocyte selection associated	-8,772	4,966E-07
Itm2a	integral membrane protein 2A	-9,346	6,535E-07
Plac8	placenta-specific 8	-9,524	3,526E-07
Slamf6	SLAM family member 6	-9,524	3,526E-07
Akr1c18	aldo-keto reductase family 1 member C18	-10,753	3,526E-07
Cpa3	carboxypeptidase A3 mast cell	-13,514	4,408E-08
Cd24a	CD24a antigen	-17,857	2,393E-08
